# Biochemical fulvic acid mediates nutrients and microbial community structure in different textured sodic saline-alkali soils

**DOI:** 10.3389/fmicb.2026.1869726

**Published:** 2026-08-18

**Authors:** Yan Sun, Bingbing Wu, Bowei Ren

**Affiliations:** 1 State Key Laboratory of Eco-Hydraulics in Northwest Arid Region of China, Xi'an University of Technology, Xi'an, China; 2 Xinjiang Future Irrigation District Engineering Technology Research Center, Xinjiang, China

**Keywords:** biochemical fulvic acid, microorganisms, nutrient availability (soil), sodic saline-alkali soil, soil texture

## Abstract

**Background:**

Sodic saline-alkali soils restrict agricultural productivity by limiting nutrient availability and disrupting microbial communities, yet their responses to biochemical fulvic acid (BFA) across sodic saline-alkali soils with different textures remain unclear.

**Methods:**

A 45-day controlled incubation experiment was conducted using sandy loam and loamy sand amended with BFA at 0, 1, 2, 4, and 8 g kg^−2^.

**Results:**

Responses to BFA differed between the two soils with different textures and among application rates and were most pronounced during the early incubation period. In sandy loam, 2 g·kg^−1^ BFA increased ammonium nitrogen and nitrate nitrogen by 68% and 53%, respectively, on day 15 relative to the control. In loamy sand, 1 g·kg^−1^ BFA increased nitrate nitrogen by 92% on day 15 and ammonium nitrogen by 70% on day 30. Sequencing analysis of soil samples collected on day 45 revealed distinct microbial community responses to 2 g·kg^−1^ BFA in sandy loam and 1 g·kg^−1^ BFA in loamy sand.

**Results:**

Within the conditions of this trial, the optimum application rates were 2 g·kg^−1^ in sandy loam and 1 g·kg^−1^ in loamy sand, corresponding to the most remarkable responses.

## 1 Introduction

Soil salinization is an environmental issue of global concern, posing a serious threat to ecological and agricultural sustainability. China is one of the countries most severely affected by soil salinization and sodification, with sodic saline-alkali soils covering approximately 99.13 million hectares, accounting for 10.1% of the global sodic saline-alkali land area, and salinization of cultivated land continues to intensify in some regions (Lei et al., 2025). Advancing the comprehensive transformation and sustainable utilization of saline-alkali land holds significant strategic importance for safeguarding national food and ecological security. First, the high concentration of exchangeable Na^+^ in sodic saline-alkali soils disrupts their physicochemical properties, leading to soil compaction and poor soil structure (Barzegar et al., 1996). Second, salt concentration constitutes a stress factor for plant growth, as excessive salt ions and pH levels in the soil cause essential nutrients such as phosphorus, iron, and zinc to form insoluble precipitates. Even with increased fertilization, these nutrients remain inaccessible to plants, creating the paradoxical situation where the “soil is fertile, yet plants starve.” In contrast, salt induces osmotic stress, lowering the soil water potential below that of plant root cells. This prevents crops from absorbing water, even when moisture is abundant, leading to physiological drought and subsequent wilting (Song J. et al., 2025). Among the various sodic saline-alkali soils, sodic saline-alkali soils present particularly significant challenges for improvement owing to their combination of extremely high pH values and high exchangeable sodium content, resulting in poor soil structural stability. This makes them one of the major challenges currently facing the improvement and utilization of sodic saline-alkali land (Rengasamy, 2002). Sodic saline-alkali soils in China are primarily distributed in the arid regions of the northwest and the Songnen Plain, forming a significant component of sodic saline-alkali land. These areas constitute vital portions of both arable land and grasslands, and hold immense importance for national food security and ecological safety. Based on the realities of agricultural development in China, exploring green management approaches for sodic saline-alkali soils that balance food security with sustainable arable land use is a critical task for safeguarding the “1.8 billion mu arable land red line” of China and advancing green and sustainable agricultural development (Lei et al., 2025).

Soil microorganisms are indispensable components of soil ecosystems and are closely related to soil fertility (Van der Heijden et al., 2008). They participate in processes such as soil nutrient cycling and energy transfer and play a vital role in maintaining soil functionality. However, soil salinization significantly suppresses microbial activity, reduces community richness and diversity, and substantially alters the structure and function of the microbial community. In turn, this affects soil fertility, nutrient cycling, and plant growth (Li et al., 2024; Wichern et al., 2006). The detrimental effects of sodic saline-alkali soil on microbial communities are primarily manifested in the following aspects. First, high salt concentrations impair the growth and metabolic activity of soil microorganisms through osmotic stress and ionic toxicity, thereby reducing microbial activity (Rath et al., 2016). Second, sodic saline-alkali stress inhibits the metabolic activity of most bacteria and fungi in the soil, allowing only a few salt-tolerant microorganisms to survive. This leads to a significant reduction in the abundances of functional microbial communities involved in organic matter decomposition, nitrification, and nitrogen fixation, resulting in decreased microbial community diversity. Ultimately, the microbial community structure becomes simplified, and ecological functions decline (Zhang et al., 2024; Li et al., 2021). Furthermore, disruption of microbial communities leads to a decline in soil ecological functions and impedes nutrient cycling, making it difficult to support agricultural production and vegetation growth. Among the current sodic saline-alkali land remediation measures, physical remediation methods (such as deep plowing and subsurface drainage) can improve soil aeration and salt distribution to some extent. However, under certain conditions, they may also disrupt the original soil aggregate structure and alter the microbial community composition, thereby adversely affecting the soil ecological environment (Cania et al., 2019). Chemical amendment measures (such as gypsum and desulfurization gypsum) can lower soil pH and improve its physicochemical properties to some extent by replacing exchangeable Na^+^ in the soil. However, they have the drawbacks of high amendment costs and a tendency to cause secondary pollution (Wang J. et al., 2023). Biological improvement measures do not involve forcibly altering the environment through purely physical or chemical means. Instead, they introduce organic substances, such as humic and fulvic acids, to provide carbon sources and energy for microorganisms. This promotes the proliferation of beneficial microbes and optimizes microbial community structures, thereby enhancing soil fertility, increasing soil biodiversity, and strengthening ecological functions (Meng et al., 2025; Song Q. et al., 2025). Therefore, it is widely regarded as one of the most ecologically friendly and sustainable approaches for the treatment of sodic saline-alkali land.

Various organic and inorganic amendments have been used to improve sodic saline-alkali soils, each with distinct effects. Humic acid and humin mainly enhance soil structure, nutrient retention, and long-term carbon stability (Nardi et al., 2021), whereas inorganic amendments such as gypsum primarily reduce soil sodicity but exert limited influence on microbial activity (Gashi et al., 2025). Crop residues, compost, and biochar provide labile carbon, stimulating microbial growth and nutrient cycling (Canellas and Olivares, 2014). In contrast, biochemical fulvic acid (BFA) combines high chemical reactivity, abundant functional groups, and direct stimulation of soil microbial communities, positioning it as a particularly promising amendment for improving both the chemical and biological properties of sodic soils across different textures. Fulvic acid is a low-molecular-weight fraction of humic substances characterized by high chemical reactivity and abundant functional groups, making it a widely used and environmentally friendly soil amendment (Fragouli et al., 2023). Fulvic acid is further classified into BFA and mineral-source fulvic acid. Biochemical fulvic acid is produced from industrial and agricultural organic waste materials (such as crop straw and livestock manure) via controlled microbial fermentation. Mineral-source fulvic acid originates from mineral deposits formed during ancient geological periods and is typically extracted from weathered coal, lignite, or peat (Chi et al., 2023). In addition, BFA is a low-molecular-weight organic fraction enriched in carbon, oxygen, hydrogen, nitrogen, and sulfur, and contains abundant oxygen-containing functional groups, including carboxyl (-COOH), phenolic hydroxyl (-OH), and carbonyl groups. These functional groups confer strong metal-chelating and cation-binding capacities, enabling fulvic acid to form stable complexes with multivalent metal ions (e.g., Fe^3+^, Al^3+^, Ca^2+^, and Mg^2+^), thereby enhancing micronutrient solubility and mobility in soils (Sutton and Sposito, 2005; Stevenson, 1982). The high density of dissociable acidic groups further contributes to its electrolyte behavior in soil solution, regulating cation exchange reactions, ionic equilibrium, and buffering processes (Stevenson, 1982). Moreover, fulvic acid may interact with soil microorganisms by serving as a labile carbon substrate, modulating extracellular enzyme activity, and influencing nutrient availability, which can stimulate microbial metabolism and reshape community structure (Li et al., 2022; Zhang et al., 2020). During the remediation of sodic saline-alkali soils, BFA has been reported to regulate soil physicochemical properties and alter ion dynamics. More importantly, it may influence soil microbial communities by supplying reactive organic constituents that stimulate microbial activity and metabolism. Such microbial responses can subsequently affect soil ecological processes and nutrient cycling; however, the underlying mechanisms remain largely unclear (Xue et al., 2024).

In addition, most existing studies have mainly focused on the effects of fulvic acid on soil physicochemical properties or crop performance, whereas limited attention has been paid to its impact on soil microbial community structure, particularly in sodic saline-alkali soil with different textures. The interactive effects of soil texture, ionic dynamics, and microbial community responses under BFA application remain insufficiently understood. Clarifying these relationships is essential for optimizing BFA application strategies and improving sodic saline-alkali soil management. To investigate the ameliorative effects of BFA on sodic saline-alkali soils, we used two distinct textural types of sodic saline-alkali soils (sandy loam and loamy sand) as experimental subjects. This study examined the effects of different BFA application rates on soil physicochemical properties, nutrient availability, and microbial community structure in sodic saline–alkali soils of contrasting textures, and evaluated the associations between microbial community variation and soil environmental factors.

## 2 Materials and methods

### 2.1 Test materials

For each soil type, surface soil from the 0–20 cm layer was collected from a sodic saline–alkali site in Horqin Left Middle Banner, Inner Mongolia Autonomous Region, China (longitude 110 °10′E, latitude 40 °29′N); upper and lower portions of this layer were not sampled separately. After collection, plant residues, gravel, and other impurities were removed from each soil, which was then thoroughly homogenized, air-dried in the dark, and passed through a 2-mm sieve before incubation. Soil texture was determined using a Mastersizer 2000 laser particle size analyzer (Malvern Instruments Ltd., UK). The two soils were classified as sandy loam (74.24% sand, 21.04% silt, 4.70% clay) and loamy sand (87.53% sand, 10.41% silt, 2.05% clay). Bulk density, measured by the ring knife method, was 1.40 g·cm^−3^ for sandy loam and 1.48 g·cm^−3^ for loamy sand. Soil pH was 8.61 and 8.69, and initial salt content was 1.4539 g·kg^−1^ and 1.3216 g·kg^−1^ for sandy loam and loamy sand, respectively. Both soils are classified as mildly sodic saline-alkali. Initial salt ion concentrations are presented in Table 1. BFA was provided by Matsumoto Technology Co., Ltd. in Langfang, Shandong Province. The product was a brown powder with a pH of 5–6, water solubility ≥ 99%, and a specific surface area of 43.781 m^2^·g^−1^. The elemental composition and atomic ratios are given in Tables 2, 3, respectively.

**Table 1:** Initial salt ion concentrations of two sodic saline-alkali soils (cmol·kg^−1^).

Soil sample	K^+^	Na^+^	Ca^2+^	Mg^2+^	${\text{HCO}}_{3}^{-}$	Cl^−^	${\text{SO}}_{4}^{2-}$	${\text{CO}}_{3}^{2-}$
Sandy loam	0.019	0.191	0.371	0.096	0.517	0.072	0.161	–
Loamy sand	0.052	0.025	0.215	0.074	0.265	0.073	0.109	–

**Table 2:** Elemental composition (C, H, O, and N) of BFA.

Elemental	Content (%)
C	38.59
H	4.83
O	35.82
N	1.60

**Table 3:** Atomic ratios of BFA.

Atomic ratio	Value
H/C	1.50
O/C	0.70
N/C	0.04

### 2.2 . Experimental design

A completely randomized design comprising two soil types and five BFA application rates was used, resulting in 10 treatment combinations (Table 4). Each treatment consisted of nine independent pots. Three pots per treatment were destructively sampled on each of days 15, 30, and 45, providing three independent biological replicates per treatment at each sampling time (*n* = 3; 90 pots in total).

**Table 4:** Experimental design and treatments.

Experimental treatment	BFA application rates (g·kg^−1^)	Soil Type	Destructive sampling	Physicochemical measurements	Microbial sequencing
BF0	0	Sandy loam	Days 15, 30, and 45		Day 45
BF1	1	Sandy loam			Not sequenced
BF2	2	Sandy loam			Day 45
BF4	4	Sandy loam			Not sequenced
BF8	8	Sandy loam			Not sequenced
XBF0	0	Loamy sand			Day 45
XBF1	1	Loamy sand			Day 45
XBF2	2	Loamy sand			Not sequenced
XBF4	4	Loamy sand			Not sequenced
XBF8	8	Loamy sand			Not sequenced

Each treatment comprised nine independent pots. Three different pots were destructively sampled on days 15, 30, and 45, providing three biological replicates per treatment at each sampling time (n = 3). Physicochemical measurements included pH, EC, ${\text{NH}}_{4^{+}}$-N, ${\text{NO}}_{3^{-}}$-N, AP, AK, Ca^2+^ and Mg^2+^. Microbial sequencing was conducted only for BF0, BF2, XBF0, and XBF1 using samples collected on day 45.

The bare-soil incubation experiment was conducted in a controlled-environment greenhouse at Xi'an University of Technology, China. Plastic pots with an upper diameter of 14 cm, a lower diameter of 9 cm, and a height of 15 cm were randomly arranged in the greenhouse. The temperature and relative humidity were maintained at 25 °C and 50%, respectively. The light intensity was set at 40 klux, with a 10-h photoperiod from 08:00 to 18:00. During incubation, 200 mL of water was applied to each pot every other day. The start of incubation was designated as day 0, and soil samples were collected on days 15, 30, and 45. At each sampling time, each soil sample was divided into three portions. One fresh portion was used promptly for the determination of ${\text{NH}}_{4^{+}}$-N, ${\text{NO}}_{3^{-}}$-N; a second portion was air-dried and passed through a 2-mm sieve for the other physicochemical analyses; and a third portion was immediately stored at −80 °C for DNA extraction and microbial community analysis.

### 2.3 Indicator measurement

#### 2.3.1 Determination of soil physicochemical properties

Except for the determination of ${\text{NH}}_{4^{+}}$-N, ${\text{NO}}_{3^{-}}$-N, for which fresh soil was used, soil samples were air-dried and passed through a 2-mm sieve before analysis. Soil pH was measured in a 1:2.5 soil-to-water suspension using a PHSJ-5T laboratory pH meter (Shanghai INESA Scientific Instrument Co., Ltd., China). Soil electrical conductivity (EC) was determined by extracting 18 g of air-dried soil with 90 mL of deionized water at a soil-to-water ratio of 1:5. After thorough mixing and equilibration for 8 h, EC was measured using a DDS-307 conductivity meter (Shanghai INESA Scientific Instrument Co., Ltd., China; Sparks et al., 1996). For the determination of ammonium nitrogen (${\text{NH}}_{4^{+}}$-N) and nitrate nitrogen (${\text{NO}}_{3^{-}}$-N), 4 g of fresh soil was extracted with 40 mL of 1 mol·L^−1^ KCl. The suspension was shaken for 60 min, allowed to settle, and filtered, and the concentrations of ${\text{NH}}_{4^{+}}$-N, ${\text{NO}}_{3^{-}}$-N in the filtrate were determined using a SmartChem 450 automated discrete analyzer (AMS Alliance, Italy; Sparks et al., 1996). Available phosphorus (AP) was extracted from 2 g of soil with 50 mL of 0.5 mol·L^−1^ NaHCO_3_ for 30 min and determined by the molybdenum–antimony colorimetric method using a UV–visible spectrophotometer (JASCO Corporation, Japan; Olsen et al., 1954). Available potassium (AK) was extracted from 4 g of soil with 40 mL of 1 mol·L^−1^ NH_4_OAc for 30 min and determined using a flame photometer (Beijing Eastwest Analytical Instruments Co., Ltd., China; Sparks et al., 1996). Exchangeable Ca^2+^ and Mg^2+^ were extracted from 2.00 g of air-dried soil with 50 mL of 1 mol·L^−1^ NH_4_OAc adjusted to pH 7.0. The suspension was shaken for 30 min and filtered, and Ca^2+^ and Mg^2+^ concentrations in the extract were quantified using an AA-7003 atomic absorption spectrometer (Beijing Eastwest Analytical Instruments Co., Ltd., China; Sparks et al., 1996). Calibration curves prepared from the corresponding standard solutions were used for instrumental quantification.

#### 2.3.2 High-throughput sequencing of soil microorganisms

Total microbial DNA was extracted from each soil sample using the Quick-DNA Fecal Kit (ZYMO Research, USA). DNA concentration and purity were assessed using an Epoch microplate spectrophotometer (BioTek, USA), and qualified DNA samples were stored at −80 °C until use. For bacterial community analysis, the V4 region of the 16S rRNA gene was amplified using the barcode-tagged primers 515F (5′-GTGCCAGCMGCCGCGGTAA-3′) and 806R (5′-GGACTACHVGGGTWTCTAAT-3′). For fungal community analysis, the ITS1 region was amplified using the barcode-tagged primers ITS5-1737F (5′-GGAAGTAAAAGTCGTAACAAGG-3′) and ITS2-2043R (5′-GCTGCGTTCTTCATCGATGC-3′). Each 15-μL PCR reaction contained Phusion High-Fidelity PCR Master Mix (New England Biolabs, USA), 0.2 μM of each forward and reverse primer, and approximately 10 ng of template DNA. The thermal-cycling conditions consisted of initial denaturation at 98 °C for 1 min, followed by 30 cycles of denaturation at 98 °C for 10 s, annealing at 50 °C for 30 s, and extension at 72 °C for 30 s, with a final extension at 72 °C for 5 min. PCR products were examined by 2% agarose gel electrophoresis, pooled in equimolar amounts, and purified. Indexed sequencing libraries were subsequently constructed. Library fragment integrity and insert-size distributions were assessed using an automated capillary electrophoresis system (AATI), and effective library concentrations were quantified by real-time PCR. Qualified libraries were pooled and subjected to paired-end sequencing (2 × 250 bp) on an Illumina NovaSeq platform.

### 2.4 Bioinformatics and statistical analysis

**Figure 1:**
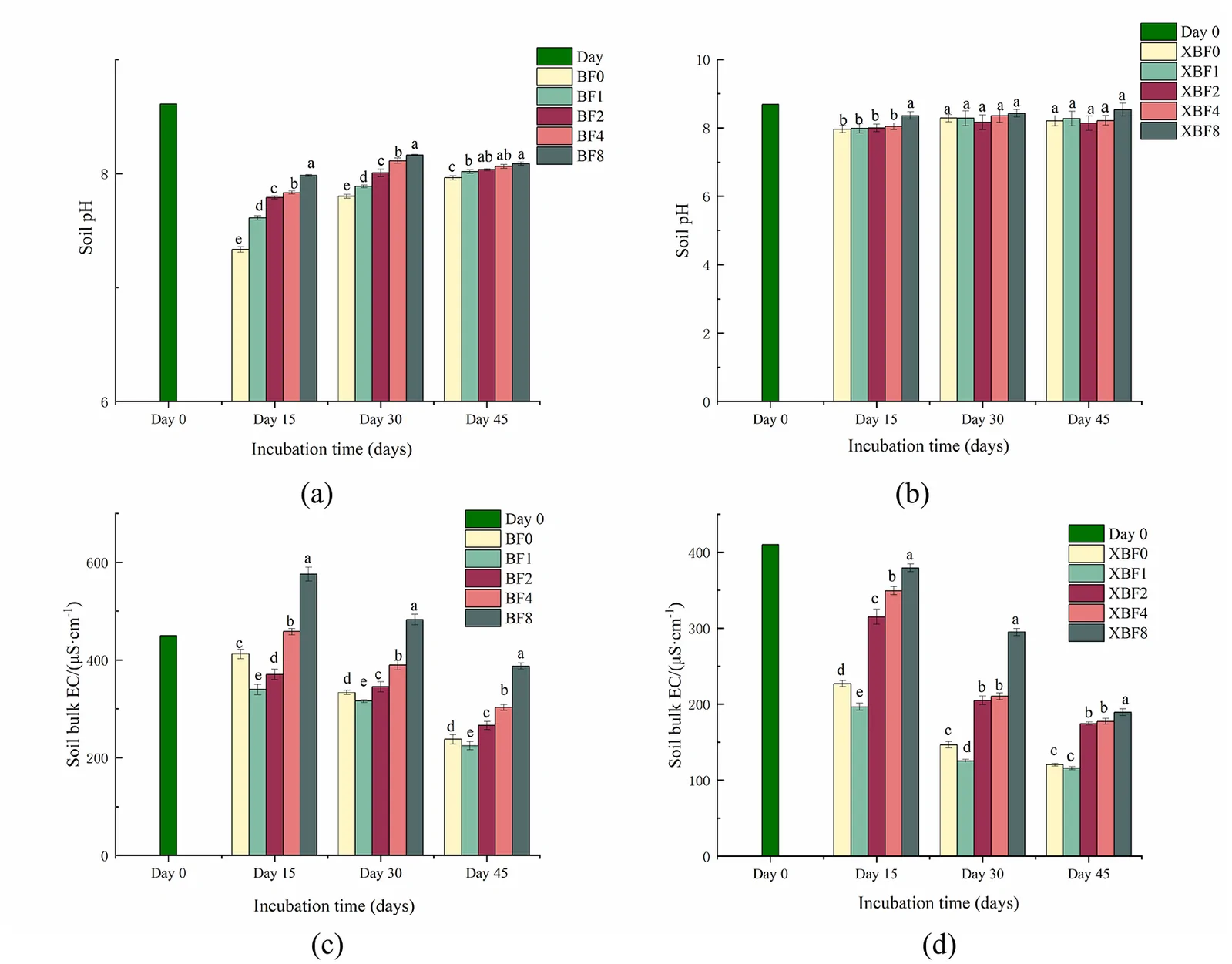
Effects of different biochemical fulvic acid (BFA) application rates on soil pH and electrical conductivity (EC) during incubation. **(a)** Soil pH in sandy loam; **(b)** soil pH in loamy sand; **(c)** soil EC in sandy loam; **(d)** soil EC in loamy sand. The dark green bars at day 0 represent the initial values before BFA application. Values are means ± standard deviation (SD) of three independent biological replicates (*n* = 3). Within the same soil type and sampling time, different lowercase letters above the bars indicate significant differences among BFA application rates according to Fisher's protected least significant difference (LSD) test following a significant overall ANOVA (*P* < 0.05), whereas bars sharing at least one letter are not significantly different.

**Figure 2:**
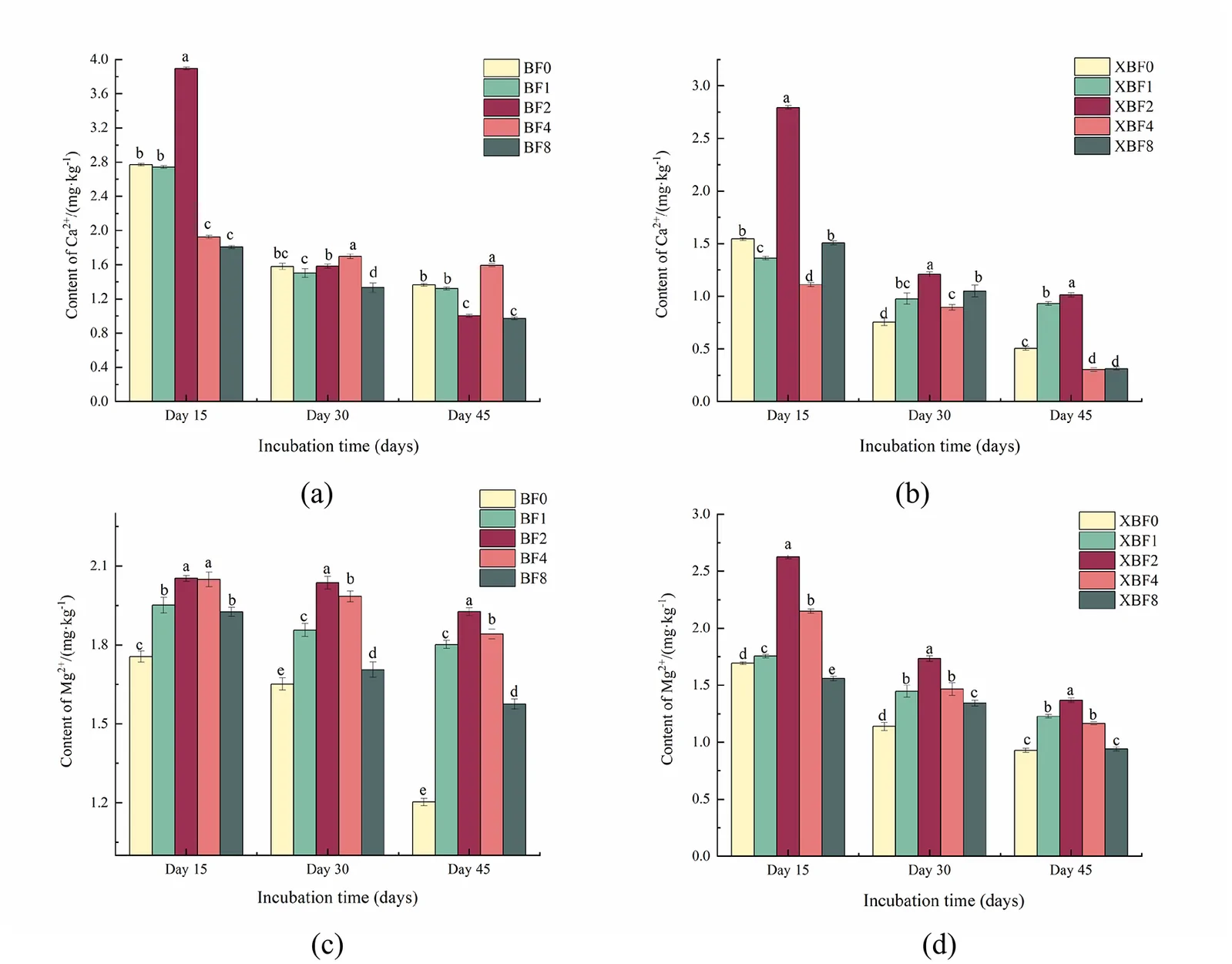
Effects of different biochemical fulvic acid (BFA) application rates on soil calcium (Ca^2+^) and magnesium (Mg^2+^) concentrations during incubation. **(a)** Ca^2+^ concentration in sandy loam; **(b)** Ca^2+^ concentration in loamy sand; **(c)** Mg^2+^ concentration in sandy loam; **(d)** Mg^2+^ concentration in loamy sand. Values are means ± standard deviation (SD) of three independent biological replicates (*n* = 3). Within the same soil type and sampling time, different lowercase letters above the bars indicate significant differences among BFA application rates according to Fisher's protected least significant difference (LSD) test following a significant overall ANOVA (*P* < 0.05), whereas bars sharing at least one letter are not significantly different.

**Figure 3:**
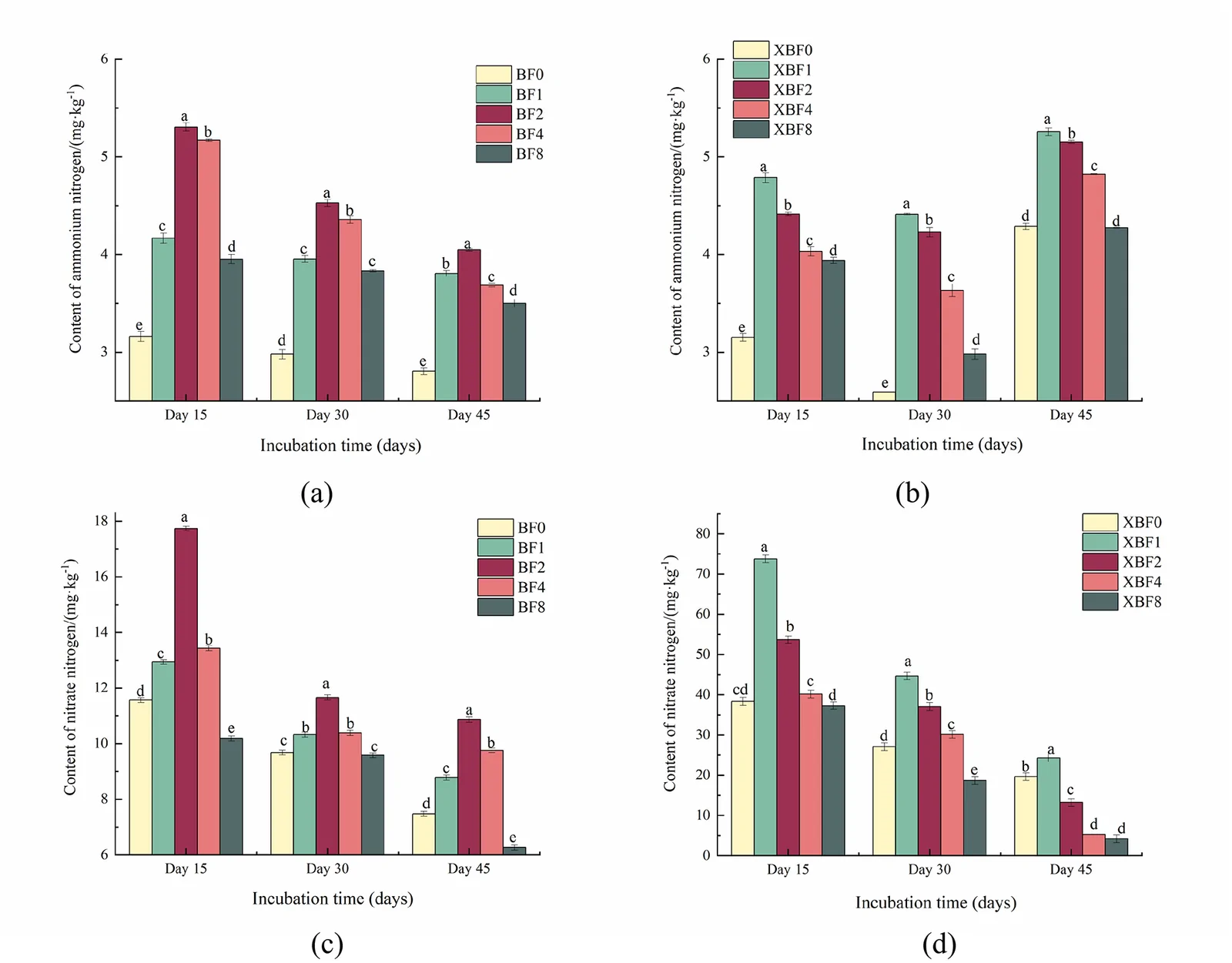
Effects of different biochemical fulvic acid (BFA) application rates on soil ammonium nitrogen (${\text{NH}}_{4^{+}}$-N) and nitrate nitrogen (${\text{NO}}_{3^{-}}$-N) concentrations during incubation. **(a)** ${\text{NH}}_{4^{+}}$-N concentration in sandy loam; **(b)** ${\text{NH}}_{4^{+}}$-N concentration in loamy sand; **(c)** ${\text{NO}}_{3^{-}}$-N concentration in sandy loam; **(d)** ${\text{NO}}_{3^{-}}$-N concentration in loamy sand. Values are means ± standard deviation (SD) of three independent biological replicates (*n* = 3). Within the same soil type and sampling time, different lowercase letters above the bars indicate significant differences among BFA application rates according to Fisher's protected least significant difference (LSD) test following a significant overall ANOVA (*P* < 0.05), whereas bars sharing at least one letter are not significantly different.

**Figure 4:**
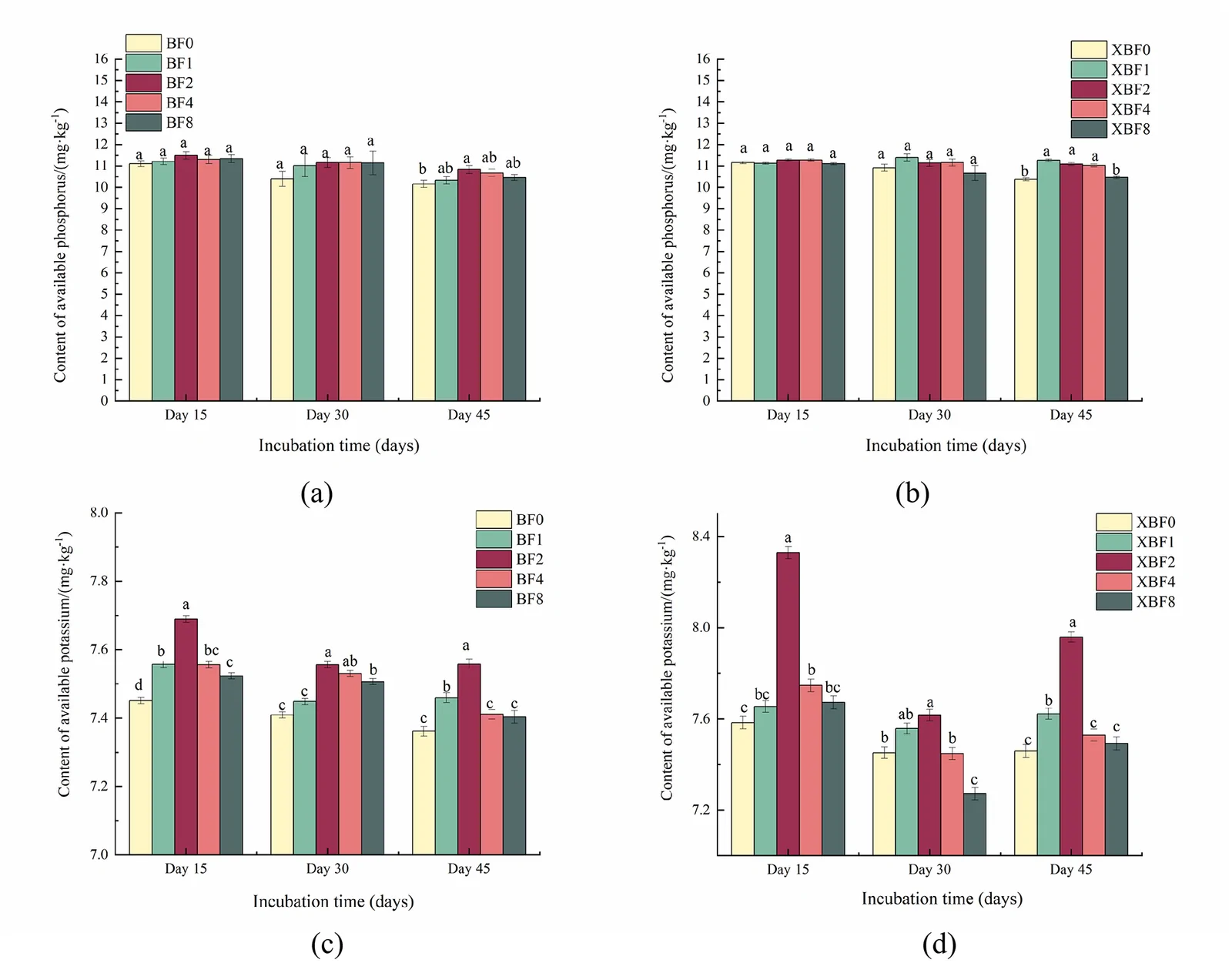
Effects of different biochemical fulvic acid (BFA) application rates on soil available phosphorus (AP) and available potassium (AK) concentrations during incubation. **(a)** AP concentration in sandy loam; **(b)** AP concentration in loamy sand; **(c)** AK concentration in sandy loam; **(d)** AK concentration in loamy sand. Values are means ± standard deviation (SD) of three independent biological replicates (*n* = 3). Within the same soil type and sampling time, different lowercase letters above the bars indicate significant differences among BFA application rates according to Fisher's protected least significant difference (LSD) test following a significant overall ANOVA (*P* < 0.05), whereas bars sharing at least one letter are not significantly different.

Paired-end reads were assigned to individual samples according to their unique barcodes, and the barcode and primer sequences were subsequently removed. The reads were merged using FLASH (v1.2.11), quality-filtered using fastp (v0.23.1), and screened for chimeras using the UCHIME algorithm with the SILVA reference database for bacterial 16S rRNA gene sequences and the UNITE reference database for fungal ITS sequences. The resulting effective tags were denoised using the DADA2 module in QIIME 2 (release 2022.2) to generate amplicon sequence variants (ASVs). Taxonomic assignment was performed in QIIME 2 using SILVA 138.1 for bacterial 16S rRNA gene sequences and UNITE for fungal ITS sequences. The ASV abundance tables were normalized to the sequencing depth of the sample with the fewest sequences before subsequent analyses. Alpha diversity was evaluated using observed ASVs and the Chao1, Shannon, and Simpson indices. Microbial community patterns were visualized by principal component analysis (PCA) of the normalized ASV abundance data using the ade4 and ggplot2 packages in R. Because PCA was performed directly on the normalized abundance matrix, no separate distance matrix was used. Heatmaps of the 35 most abundant genera were generated using the pheatmap package in R. Physicochemical data are presented as the mean ± standard deviation (SD) of three independent biological replicates (*n* = 3). Because different pots were destructively sampled on days 15, 30, and 45, observations from the three sampling times were independent and were not treated as repeated measurements. Statistical analyses of soil physicochemical properties and the corresponding graphing were performed using OriginPro 2024, whereas Microsoft Excel 2021 was used for data organization. For each soil type and sampling time, the effects of BFA application rate on soil physicochemical properties were evaluated separately using one-way analysis of variance (ANOVA). When the overall ANOVA was significant, pairwise differences among the five BFA application rates were evaluated using Fisher's protected least significant difference (LSD) test at *P* < 0.05. Statistical letters in Figures 1–4 therefore represent comparisons among BFA application rates within the same soil type and sampling time. Day 0 values were used only as initial baselines and were not included in comparisons among BFA treatments. Microbial community analyses were conducted using only the selected day 45 samples. Alpha-diversity indices were analyzed separately for each soil type using one-way ANOVA to compare the control and selected BFA treatment, with *P* < 0.05 considered statistically significant. Differential taxa were identified using linear discriminant analysis effect size (LEfSe), with a Kruskal–Wallis test at *P* < 0.05 and an LDA score > 4.0. Before RDA, microbial abundance data were Hellinger-transformed. RDA was performed using the vegan package (v2.6-4) in R (v4.3.1), with all measured soil physicochemical variables included as constraining variables. The significance of the overall RDA model and individual constrained axes was assessed using 999 permutations, and environmental vectors were fitted using envfit with 999 permutations. AP and AK were retained in the RDA model but omitted from the plotted environmental vectors for visual clarity. Mantel tests were performed using Pearson correlation, and the resulting *P* values were adjusted using the Bonferroni method to account for multiple testing. Microbial co-occurrence networks at the phylum and genus levels were constructed using Spearman's rank correlations, and only correlations with |r| > 0.7 and *P* < 0.05 were retained.

## 3 Results and discussion

### 3.1 Effects of biochemical fulvic acid on two types of soil physicochemical properties

#### 3.1.1 Soil pH and soil electrical conductivity

Soil pH is a key indicator of soil acidity and alkalinity. It significantly influences soil physicochemical properties and fertility and serves as an important reference indicator for evaluating the effectiveness of sodic saline-alkali soil remediation. As shown by the initial baseline (day 0), both the sandy loam and loamy sand exhibited a relatively high initial pH. Following the initiation of the incubation, the pH of all treatment groups generally decreased compared to the initial day 0 levels, likely due to natural soil buffering responses, moisture changes, or early microbial respiration during the cultivation process. Despite this overall initial decrease, soil pH differed among BFA application rates within individual sampling times, and the magnitude of these differences changed during incubation (Figure 1a). Compared with CK (control), the BF1, BF2, BF4, and BF8 treatments all significantly increased soil pH at both 15 and 30 d (*P* < 0.05), effectively mitigating the pH drop; on day 15, the pH values were approximately 4%, 6%, 7%, and 9% higher than those of CK, respectively, and on day 30, values were approximately 1%, 3%, 4%, and 5% higher, respectively. All differences were significant (*P* < 0.05). In sandy loam, the magnitude of the pH response decreased over time; however, the pH values of BF1, BF2, BF4, and BF8 remained significantly higher than that of BF0 on day 45 (*P* < 0.05). In the loamy sand soil (Figure 1b), the overall decrease in pH from day 0 was less drastic, but a similar counteracting effect from BFA was less pronounced. The XBF8 treatment generally showed the highest pH values during the incubation period. During the early cultivation phase, pH changes in the other treatment groups showed no significant differences. In the later cultivation phase, the XBF2 treatment had the lowest pH (8.13-8.17). In the later cultivation phase, the differences among treatments became smaller and were no longer significant. At the same sampling time and BFA application rate, the two soils showed different pH response patterns, indicating soil-specific responses to BFA application. However, both soil types showed higher pH values than their respective controls during the early incubation stage, although this effect was more evident in sandy loam than in loamy sand. This may be attributed to the introduction of soluble alkaline constituents with the BFA, including bicarbonate if present, which may have increased soil-solution alkalinity and partially offset the pH decline. This effect became more pronounced with increasing BFA dosage. Furthermore, Ratzke and Gore (2018) indicated that microbial metabolic activity influences the pH of the local environment. Therefore, the initially higher soil pH observed in the BFA-treated soils relative to the control may be attributed to the combined effects of BFA-induced changes in soil chemistry and microbial activity during the early incubation stage. Over time, continued water movement, ion redistribution, and soil buffering reduced the magnitude of the BFA effect. By day 45, significant treatment differences persisted in sandy loam but were no longer observed in loamy sand, indicating soil-specific differences in the persistence of the pH response. Similar pH dynamics associated with fulvic-acid addition have been observed in other soil incubation studies. For example, Pan et al. (2020) reported that fulvic acid affected soil pH buffering capacity in acidic soils, resulting in increased pH during incubation, indicating that fulvic acid can influence soil acid-base chemistry under certain conditions.

Electrical conductivity is an indicator used to characterize soluble salt content. In the sandy loam soil (Figure 1c), the EC values of the low-concentration BF1 treatment consistently remained lower than those of CK, decreasing by 18%, 5%, and 5% on days 15, 30, and 45, respectively, indicating a further enhancement of the decline relative to the control. Conversely, the EC values of the BF2, BF4, and BF8 treatments were generally higher than the control during the incubation period, with BF4 and BF8 showing the most pronounced increases. At day 45, BF4 and BF8 increased EC by approximately 33% and 63%, respectively, compared with CK. Notably, at day 15, the high-dose BF8 treatment even temporarily exceeded the initial day 0 baseline. In the case of loamy sand soil (Figure 1d), the overall drop in EC from day 0 was more drastic across all groups. The conductivity of the low-concentration treatment (XBF1) was lower than that of the control, with decreases of 13%, 14%, and 4% at 15, 30, and 45 d, respectively; treatments XBF2 and higher doses exhibited significantly higher conductivity values than CK at all sampling periods (*P* < 0.05), with XBF8 showing the greatest increase of 67%, 101%, and 58% at days 15, 30, and 45, respectively. The observed dose-dependent and texture-dependent EC responses to BFA addition are consistent with recent findings by Valenciano et al. (2024), who reported that humic acid application altered soil electrical conductivity in a concentration-dependent manner, with effects varying significantly across soil types due to differences in buffering capacity and texture. In both soil types, the EC decreased with lower BFA application rates. This decrease likely occurs because BFA reacts with ions in the soil, thereby reducing the number of free ions in the soil (Christl et al., 2001), consequently lowering the soil's electrical conductivity and enhancing the natural decline observed from day 0. Conversely, higher application rates can elevate EC because BFA itself carries a large number of anions and cations; increased application rates can lead to rapid accumulation of exogenous ions in the soil, and various chemical reactions between BFA and existing ions in the soil may also promote an increase in soluble salt content, thereby markedly elevating EC relative to the control. By day 45, the differences in EC between treatments in sandy loam were more pronounced than those in loamy sand. This disparity may primarily stem from the higher sand content and greater permeability of the loamy sand soil, which facilitates prolonged water infiltration. This process promotes the downward migration of salts, thereby reducing the variation observed between treatments and leading to the lower overall EC compared to the initial day 0 level. In contrast, sandy loam soil has a higher proportion of fine particles, resulting in slower infiltration rates and reduced salt leaching. Consequently, the differences in EC between treatments were more pronounced.

#### 3.1.2 Soil nutrients

As a cofactor in soil biochemical reactions, magnesium ion (Mg^2+^) plays a crucial role in maintaining soil structural stability and driving microbial metabolic dynamics. Calcium ion (Ca^2+^) enhances cohesion between soil particles, improving soil structure. Therefore, the present study focused on analyzing these calcium and magnesium ions.

The calcium content in all treatments generally peaked on day 15 (Figures 2a, b) and then decreased significantly, reaching its lowest level by day 45. This change indicates that leaching loss of calcium ions may have occurred during cultivation or that insoluble complexes may have formed with soil colloids and organic matter. As shown in Figures 2c, d, the application of BFA significantly regulated the Mg^2+^ concentration in both soil types. Overall, the concentration of Mg^2+^ in the soil exhibited a trend of first increasing and then decreasing with increasing BFA application rate. The BF2 and XBF2 treatments in both soil types had the greatest effect on increasing the Mg^2+^ concentration. Compared to CK, Mg^2+^ levels in BF2 increased by 17%, 23%, and 60% on days 15, 30, and 45, respectively, and with the XBF2 treatment, the values increased by 55%, 52%, and 48%, respectively, with significant differences observed among all treatments (*P* < 0.05).

These changes may be closely related to the chemical properties of BFA. Owing to the excellent adsorption and ion exchange properties of BFA, applying it in appropriate amounts enables it to undergo displacement reactions with cations in the soil (such as Ca^2+^ and H^+^), thereby releasing Mg^2+^. Consequently, the Mg^2+^ content in the soil increases. However, under excessive BFA application, changes in soil chemical conditions and cation interactions may be associated with the observed decline in Mg^2+^ concentration. Therefore, the application rate of BFA should be carefully controlled to avoid negative effects on soil environment and crop growth.

Nitrate nitrogen and ammonium nitrogen, which are nitrogen sources directly available for plant uptake, serve as key indicators of soil fertility. As shown in Figure 3, in sandy loam (Figures 3a, c), compared with the CK treatment, the ammonium and nitrate nitrogen levels in the BF2 treatment increased the most significantly at day 15, with increases of 68% and 53%, respectively, whereas in loamy sand soil (Figures 3b, d), the ammonium nitrogen content under the XBF1 treatment showed the greatest increase at 30 d, rising by 70% compared to that in CK. Nitrate nitrogen content exhibited the highest increase on day 15, increasing by 92% relative to that of CK. Overall, BFA altered soil ${\text{NH}}_{4^{+}}$-N and ${\text{NO}}_{3^{-}}$-N concentrations, with the largest increases generally occurring at moderate application rates during the early incubation stage. Similar short-term increases in inorganic nitrogen following humic or fulvic acid application have been documented in previous studies. For instance, Canellas and Olivares (Canellas and Olivares, 2014) reported that humic substances can stimulate microbial activity and modulate soil nitrogen transformations. Moreover, organic amendments have been shown to simultaneously stimulate nitrogen mineralization and microbial immobilization, resulting in dynamic changes in soil inorganic nitrogen pools (Breza and Grandy, 2025). In sandy loam and loamy sand soils, both nitrate and ammonium nitrogen levels showed an initial increase, followed by a decrease as BFA application rates increased, with levels gradually declining over time. This change may stem from several factors. First, BFA itself contains nitrogen; when applied to soil, it decomposes into nitrogen sources that can be utilized by microorganisms. A portion of this nitrogen is converted into nitrate and ammonium nitrogen, leading to short-term increases in the soil nitrate and ammonium nitrogen levels. Second, as noted earlier, the application of BFA significantly affected the pH of both soil types. Excessively acidic or alkaline conditions inhibit microbial activity, thereby affecting nitrogen transformation processes (Ni et al., 2023). Simultaneously, BFA can undergo complexation reactions with soil colloids and other substances (Wang et al., 2025), forming compounds that are difficult for plants to absorb, thereby reducing available nitrogen content. Finally, the excessive application of BFA can lead to elevated soil nitrogen concentrations. Under such conditions, nitrate nitrogen may leach away, whereas ammonium nitrogen can be converted into nitrate nitrogen through nitrification, resulting in a reduction in the available ammonium nitrogen. However, under certain conditions, nitrate nitrogen in the soil can be reduced through denitrification and is lost in gaseous form, thereby decreasing the soil nitrate nitrogen content. This process is recognized as one of the key pathways influencing soil nitrogen distribution in water-nitrogen migration model simulations (Wang S. et al., 2023).

Available phosphorus is a key factor in regulating soil enzyme activity, whereas readily available potassium serves as an active potassium reservoir in the soil that can be directly utilized by plants and microorganisms. Both play crucial roles in the cycling and utilization of soil nutrients. Available phosphorus and potassium responded differently to BFA application depending on soil type, application rate, and sampling time. No significant differences in AP were observed among BFA treatments in either soil on days 15 and 30. On day 45, AP was significantly higher in BF2 than in BF0 in sandy loam, whereas BF1, BF4, and BF8 did not differ significantly from BF0. In loamy sand, AP was significantly higher in XBF1, XBF2, and XBF4 than in XBF0 on day 45, whereas XBF8 did not differ significantly from the control. AK showed a clearer rate-dependent pattern, with moderate application rates generally producing higher values, whereas the highest application rate did not consistently improve AK. These results indicate that moderate BFA application enhanced AP and AK availability more consistently than the highest application rate. Biochemical fulvic acid can form chelates with metal ions such as iron, aluminum, and calcium in the soil, reducing phosphorus immobilization and thereby enhancing phosphorus availability in the soil. Moreover, the rational application of BFA helps optimize soil physicochemical properties, enhances aeration and water retention capacity (Sun et al., 2022), and contributes to the maintenance of effective phosphorus content. Additionally, BFA promotes potassium release through chelation and increases the soil cation exchange capacity, thereby enhancing the readily available potassium content in soil (Tan, 2014). Biochemical fulvic acid can also promote microbial activity by adjusting soil pH, accelerating the decomposition of organic matter or minerals, and further supplementing readily available potassium nutrients. However, excessive application of BFA can cause excessive fluctuations in soil pH, suppressing microbial activity and thereby undermining the stability of available phosphorus and readily available potassium.

### 3.2 Effects of biochemical fulvic acid on soil microbial communities

**Figure 5:**
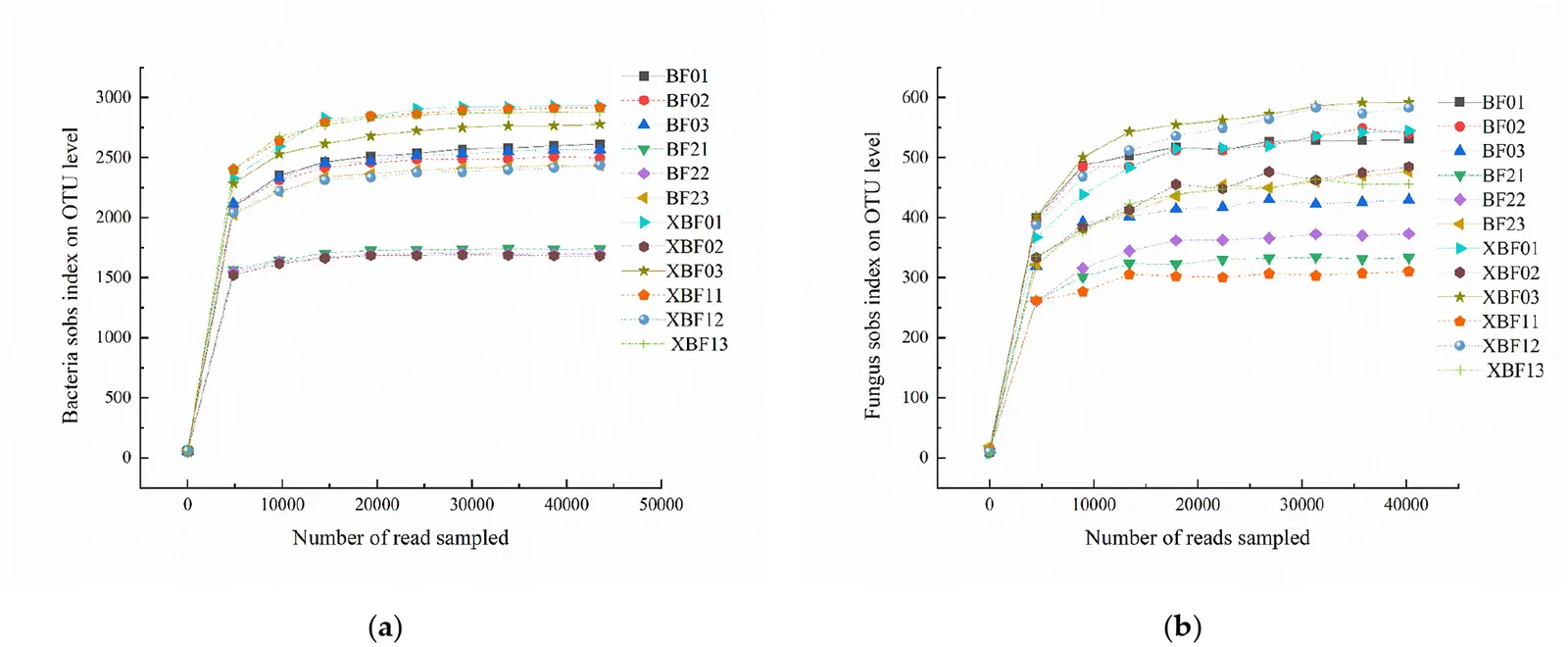
Rarefaction curves of soil bacterial and fungal communities in the selected treatments on day 45. **(a)** Bacterial communities based on 16S rRNA gene sequences; **(b)** fungal communities based on ITS sequences. Each curve represents one biological replicate. BF0 and BF2 represent sandy loam amended with 0 and 2 g·kg^−1^ BFA, respectively; XBF0 and XBF1 represent loamy sand amended with 0 and 1 g·kg^−1^ BFA, respectively. The suffixes 1–3 denote the three biological replicates of each treatment (*n* = 3).

Examination of the dilution curves for bacteria and fungi across treatments at the end of cultivation (Figures 5a, b) showed a distinct gradual flattening of the curves by day 45. Furthermore, the increase in ASV richness with increasing sequencing depth was markedly diminished, indicating that the applied sequencing coverage substantially captured the diversity of the microbial communities within the samples. The sequencing depth was considered sufficient to capture most of the observed microbial diversity, supporting subsequent community analyses.

#### 3.2.1 Microbial community diversity

**Figure 6:**
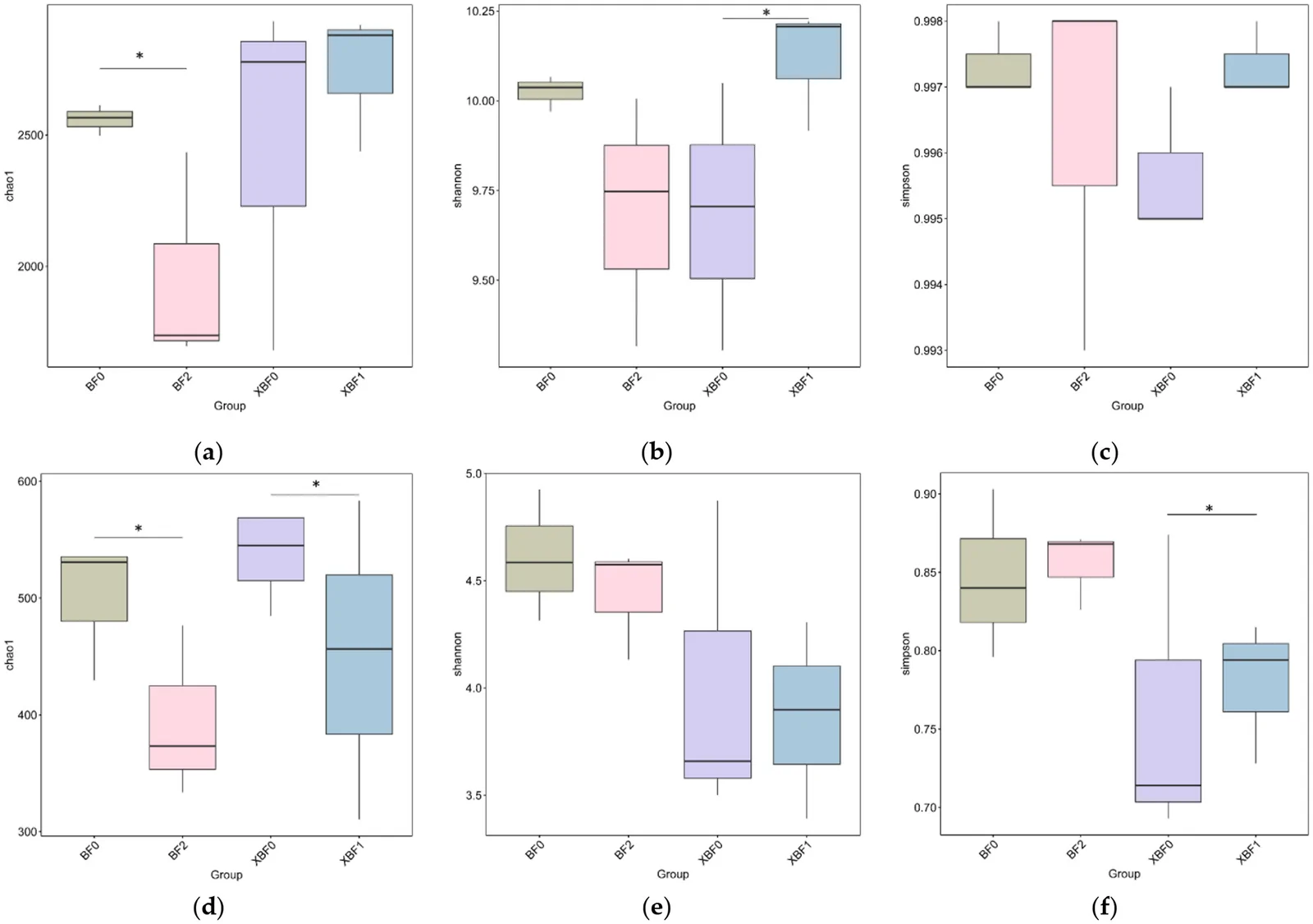
Alpha diversity of soil bacterial and fungal communities in the selected treatments on day 45. **(a–c)** Chao1, Shannon, and Simpson indices of bacterial communities, respectively; **(d–f)** Chao1, Shannon, and Simpson indices of fungal communities, respectively. BF0 and BF2 represent sandy loam amended with 0 and 2 g·kg^−1^ BFA, respectively; XBF0 and XBF1 represent loamy sand amended with 0 and 1 g·kg^−1^ BFA, respectively. Boxes represent the interquartile range, horizontal lines within boxes indicate the median, and whiskers indicate the data range (*n* = 3). Asterisks indicate significant differences between the control and BFA treatment within the same soil type (*P* < 0.05).

On day 45, high-throughput sequencing was performed on the treatments showing the most pronounced changes in soil nutrient content, namely BF0 and BF2 in sandy loam and XBF0 and XBF1 in loamy sand, with three biological replicates per treatment (*n* = 3). Figures 6a–f show the Chao1 richness and Shannon and Simpson diversity indices for bacteria and fungi in sandy loam and loamy sand soils. In sandy loam soil, compared with CK, the BF2 treatment showed significantly lower Chao1 richness indices for both fungi and bacteria (*P* < 0.05), whereas no significant differences were observed in the Shannon and Simpson indices (*P* > 0.05). The Shannon index of bacteria in loamy sand soil significantly increased (*P* < 0.05), whereas the Chao1 index was slightly higher than that of the CK treatment, but the difference was not significant (*P* > 0.05); The Chao1 and Simpson indices for fungi were both significantly reduced (*P* < 0.05). Overall, BFA tended to reduce species richness in some microbial groups while maintaining relatively stable diversity patterns in others, suggesting a restructuring rather than a uniform loss of diversity. This suggests that BFA may have altered the species composition of the communities, weakening the dominance of the previously dominant species and allowing subordinate groups to expand, thereby promoting increased community evenness.

**Figure 7:**
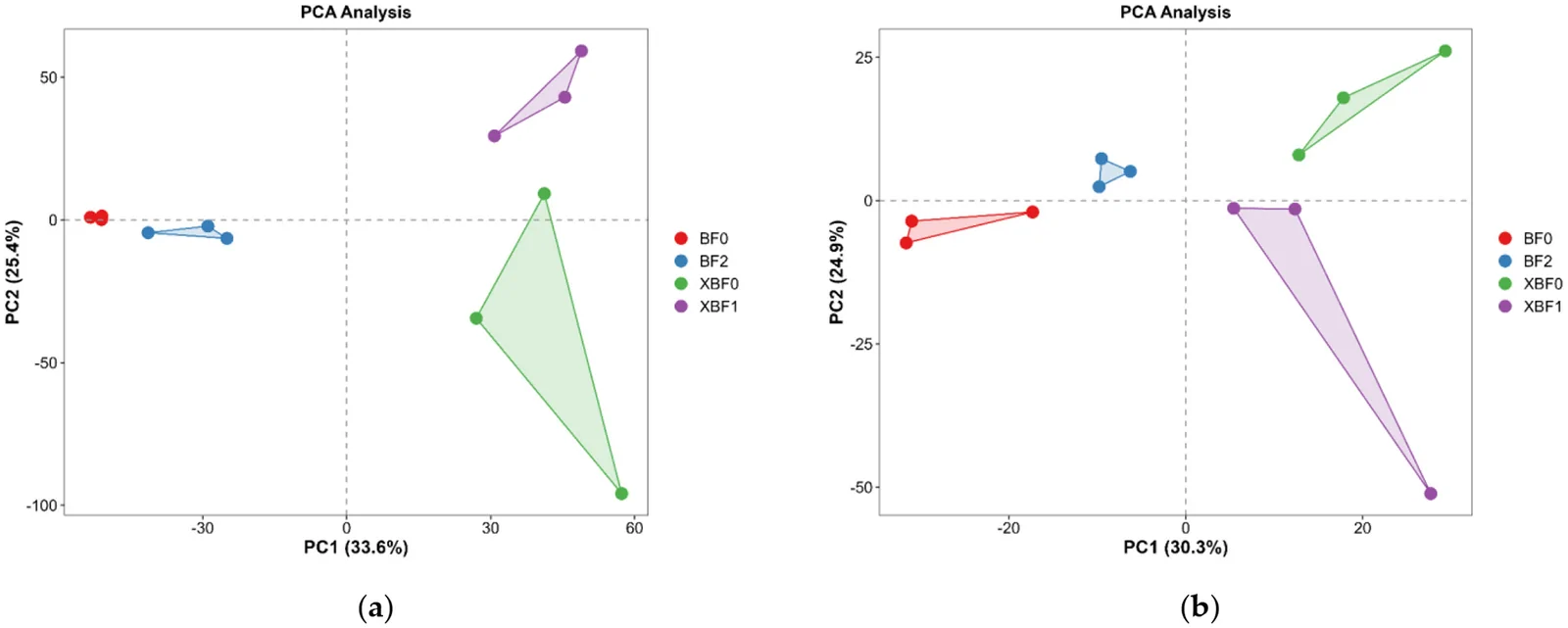
Principal component analysis (PCA) of soil bacterial and fungal community composition in the selected treatments on day 45. **(a)** Bacterial communities; **(b)** fungal communities. PCA was performed using normalized ASV abundance data. Each point represents one biological replicate, and polygons connect replicates belonging to the same treatment (*n* = 3). BF0 and BF2 represent sandy loam amended with 0 and 2 g·kg^−1^ BFA, respectively; XBF0 and XBF1 represent loamy sand amended with 0 and 1 g·kg^−1^ BFA, respectively.

Principal component analysis (PCA) was employed to visualize the two sets of samples and compare the differences in soil microbial community structure between the two groups after BFA application. The results of bacterial PCA are shown in Figure 7a. Principal components 1 and 2 accounted for 33.6% and 25.4%, respectively, of the variance. Together, they explained 59% of the variation in the microbial community structure, indicating that PCA provides relatively good representativeness. The PCA plot of fungal communities (Figure 7b) showed that principal components 1 and 2 accounted for 30.3% and 24.9%, respectively, of the variance. Together, these two components explained 55.2% of the variation in the microbial community structure, effectively reflecting the differences in community structure among treatments. The results indicated that, following BFA application, the bacterial and fungal communities in sandy loam soil showed clear separation from those in the control group, as also revealed by PCA. In loamy sand, bacterial and fungal communities showed different treatment-related patterns within their respective PCA ordinations. However, because the bacterial 16S rRNA gene and fungal ITS datasets were analyzed separately, the magnitude of separation should not be interpreted as a direct quantitative comparison between the two microbial groups. Moreover, bacteria and fungi differ in their oxygen requirements, substrate use, and sensitivity to moisture and salinity, while soil oxygen status and water content were not directly measured in this study.

#### 3.2.2 Bacterial and fungal community structure

**Figure 8:**
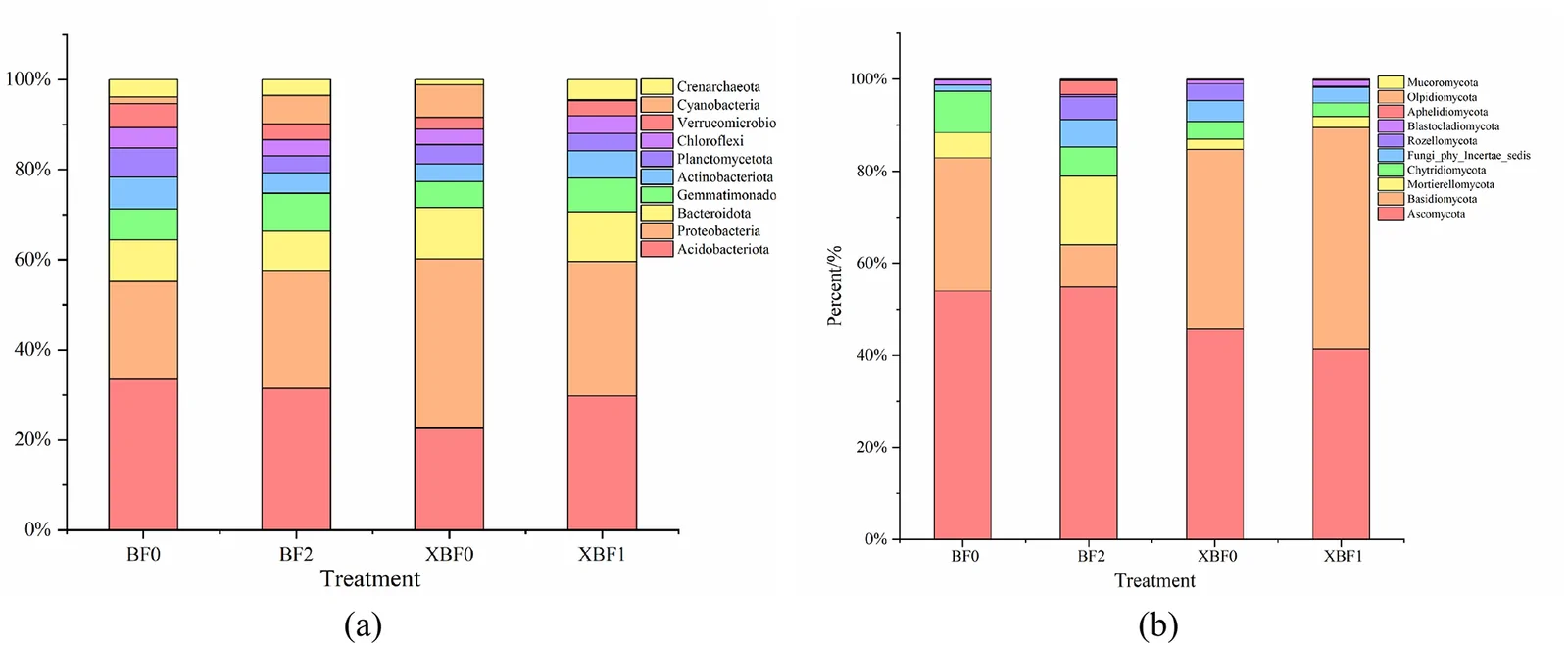
Phylum-level composition of soil bacterial and fungal communities in the selected treatments on day 45. **(a)** Relative abundance of dominant bacterial phyla; **(b)** relative abundance of dominant fungal phyla. Bars show the mean relative abundances of three independent biological replicates (*n* = 3). BF0 and BF2 represent sandy loam amended with 0 and 2 g·kg^−1^ BFA, respectively; XBF0 and XBF1 represent loamy sand amended with 0 and 1 g·kg^−1^ BFA, respectively.

High-throughput sequencing revealed differences in microbial community composition following BFA application (Figures 8a, b). At day 45, Crenarchaeota, Actinobacteriota, Proteobacteria, Planctomycetota, Bacteroidota, Chloroflexi, Gemmatimonadota, and Verrucomicrobiota were among the most abundant 16S rRNA gene taxa across treatments. Fungal communities were dominated by the phyla Basidiomycota and Ascomycota. In sandy loam soil, the application of BFA increased the relative abundances of Proteobacteria and Actinobacteriota by approximately 20% and 22%, respectively, whereas the relative abundance of Acidobacteriota decreased by approximately 9%. The relative abundance of Ascomycota within the fungal community increased by approximately 18%, whereas that of Basidiomycota decreased by approximately 12%. In loamy sand soil, the relative abundance of Proteobacteria decreased by approximately 22% in the XBF1 treatment, compared with that in the XBF0 treatment, whereas that of Acidobacteriota and Actinobacteriota increased by approximately 30% and 27%, respectively. Additionally, in loamy sand soil, the XBF1 treatment increased the relative abundance of Ascomycota (by approximately 25%), whereas that of Basidiomycota showed a slight downward trend.

In the present study, the relative abundance of Proteobacteria increased in sandy loam, indicating a positive compositional response of this phylum to BFA application under the experimental conditions. Proteobacteria are among the most metabolically active groups in soil and encompass a wide range of functional microorganisms. Research has indicated that Proteobacteria play a crucial ecological role in driving soil nutrient cycling processes (Spain et al., 2009). Moreover, many members of this phylum possess nitrogen-fixing capabilities and participate in biological nitrogen fixation in soil ecosystems. Previous studies revealed that Proteobacteria are highly sensitive to changes in soil nutrient conditions. Their abundance and community structure are frequently employed as key biological indicators of soil fertility and ecosystem response. In sandy loam, BF2 produced the strongest increases in ${\text{NH}}_{4^{+}}$-N and ${\text{NO}}_{3^{-}}$-N during the early incubation stage, and both inorganic-N forms remained significantly higher than those in BF0 on day 45. The sustained enrichment of inorganic N throughout the incubation period may have increased the availability of readily utilizable nutrients and altered competition among microbial taxa. This nutrient environment was consistent with the increased relative abundance of Proteobacteria under BF2, because many members of this phylum exhibit copiotrophic growth strategies and respond rapidly to increased availability of labile nutrients. Meanwhile, the concurrent increase in Actinobacteriota suggests that the microbial response was also shaped by organic-substrate utilization and tolerance to alkaline and saline conditions. Thus, the day 45 community composition likely represented the cumulative outcome of nutrient enrichment and soil environmental filtering during the 45-day incubation. Similar increases in Proteobacteria following humic or organic amendment application have been reported in saline soils, where enhanced nutrient availability preferentially stimulated copiotrophic bacterial groups (Song et al., 2024). Sandy loam soils inherently possess a low organic matter content and weak nutrient retention capacity (Šimanský et al., 2019). The increase in Proteobacteria relative abundance coincided with changes in inorganic nitrogen concentrations under the BF2 treatment, suggesting an association between bacterial community composition and soil nutrient conditions. This result is consistent with those of previous studies, as the Proteobacteria typically occupies a higher proportion in soils with higher organic carbon and nitrogen content and stronger microbial activity. Its relative abundance is significantly correlated with soil nutrient levels (Lauber et al., 2008). In loamy sand soil, the relative abundance of Acidobacteriota significantly increased following BFA application. Previous studies have reported that the relative abundance of Acidobacteriota is associated with changes in soil physicochemical properties following organic amendment application (Guo et al., 2022). When participating in soil carbon cycling, certain members of the phylum Acidobacteriota can also accelerate the activation and utilization of insoluble phosphorus in the soil by secreting substances such as phosphatase and organic acids, thereby enhancing phosphorus availability (Ward et al., 2009). In loamy sand, the XBF1 treatment produced a pronounced increase in ${\text{NO}}_{3^{-}}$-N on day 15 and in ${\text{NH}}_{4^{+}}$-N on day 30, with increases of 92% and 70%, respectively, relative to XBF0. By day 45, ${\text{NH}}_{4^{+}}$-N and ${\text{NO}}_{3^{-}}$-N remained significantly higher under XBF1 than under XBF0, and AP was increased by 9%. This temporal pattern indicates that BFA altered both the early nutrient-release dynamics and the nutrient status maintained during the later incubation stage. The initial inorganic-N pulse may have stimulated microbial growth and intensified competition for available substrates, whereas the sustained differences in inorganic N and AP, together with changes in pH and EC, may have progressively altered resource availability and environmental filtering. These cumulative changes provide a plausible ecological basis for the distinct microbial community composition observed under XBF1 on day 45. The increase in Acidobacteriota, Actinobacteriota, and Ascomycota and the decrease in Proteobacteria therefore likely reflected the combined effects of nutrient dynamics and the saline–alkaline soil environment, rather than a response to a single nutrient variable. Ascomycota play a vital role in soil ecosystems. Not only do they participate in soil carbon cycling but also promote the decomposition of complex organic matter by secreting various degradative enzymes, thereby accelerating nutrient cycling and organic matter mineralization (Manici et al., 2024). Certain members of the phylum Ascomycota can form symbiotic relationships with other microorganisms and plant root systems, thereby enhancing plant nutrient absorption capacity and improving stress resistance (Ramatsitsi and Manyevere, 2025). In both sandy loam and loamy sand, the relative abundance of Ascomycota increased under BFA application, suggesting a potential link between this phylum and the altered soil environment. Whether such taxonomic shifts translate into enhanced nutrient cycling or ecosystem stability, however, warrants further functional investigation.

**Figure 9:**
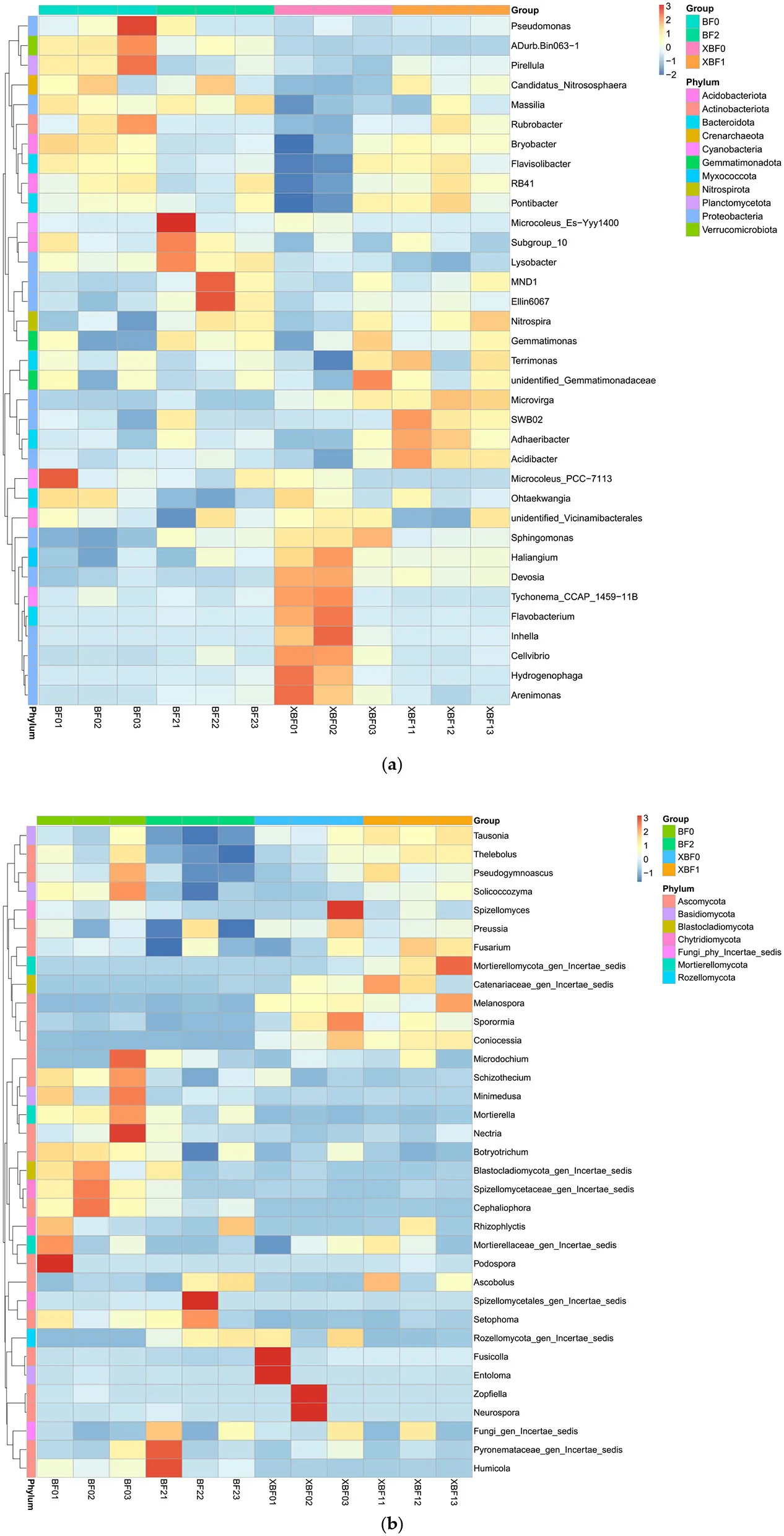
Heatmaps of the dominant bacterial and fungal genera in the selected treatments on day 45. **(a)** Top 35 bacterial genera; **(b)** top 35 fungal genera. Colors represent standardized relative abundances, with warmer colors indicating higher relative abundance and cooler colors indicating lower relative abundance. Dendrograms indicate hierarchical clustering of genera. BF0 and BF2 represent sandy loam amended with 0 and 2 g·kg^−1^ BFA, respectively; XBF0 and XBF1 represent loamy sand amended with 0 and 1 g·kg^−1^ BFA, respectively. The suffixes 1–3 denote biological replicates (*n* = 3).

The relative abundances of the two bacterial groups in the soil, using the top 35 genera, was analyzed after BFA application (Figure 9a). In sandy loam soil, following BFA application, the dominant microbial community shifted from *Pseudomonas, Massilia, Rubrobacter, Candidatus Nitrososphaera*, and others to *Microcoleus Es-Yyy1400, Lysobacter, Ellin6067, Subgroup 10*, and others. *Microcoleus Es-Yyy1400* belongs to the typical crust-forming microorganisms of Cyanobacteria, possessing functions of photosynthesis, nitrogen fixation, and soil structure stabilization, and may play a positive role in ecological restoration (Belnap et al., 2001). *Lysobacter* is commonly recognized for its antagonistic potential. Studies have revealed its relatively high abundance in healthy rhizosphere soils, where it correlates with pathogen suppression and favorable soil microbial conditions. Consequently, it is regarded as a potential indicator of soil health (Zhu et al., 2021). This shift indicates that BFA application was associated with changes in the relative abundances of several dominant bacterial genera in sandy loam. Similar shifts in soil bacterial community composition following organic amendment application have been observed in saline and sodic saline-alkali soils, where organic inputs significantly altered the relative abundances of dominant taxa and reshaped microbial community structure (Guo et al., 2022). Sandy loam has a loose texture and low organic matter content, making microorganisms susceptible to nutrient and moisture limitation. Biochemical fulvic acid can serve as a carbon source, improving the microbial environment and providing a nutritional foundation for photosynthetic and nitrogen-fixing cyanobacteria, as well as stress-resistant antagonistic bacteria. This enhances the soil stability and self-repair capacity of the ecosystem. After BFA application, in the loamy sand soil, compared to CK, the dominant microbial communities shifted from *Sphingomonas, Flavobacterium, Hydrogenophaga, Cellvibrio*, and *Tychonema* to *Nitrospira, Gemmatiomonas, Terrimonas, Acidibacter, SWB02*, and *Microvirga*. *Nitrospira* is a nitrifying bacterium that plays a crucial role in soil nitrogen cycling. It maintains the integrity of the nitrogen transformation process by oxidizing nitrite to nitrate (Daims et al., 2015). The enrichment of Nitrospira was consistent with the higher ${\text{NO}}_{3^{-}}$-N concentration under the XBF1 treatment; however, nitrification activity was not directly measured. Combined with previous results, the XBF1 treatment significantly increased the nitrate nitrogen content in loamy sand soil. This suggests that the abundant organic carbon sources in BFA may have improved the microbial habitat, thereby promoting the growth of the functional microbial communities involved in nitrogen transformation. The relative abundances of several dominant fungal genera changed in sandy loam after BFA application (Figure 9b). Because fungal functional guilds were not inferred in this study, these compositional changes should not be interpreted as evidence of a decrease in saprophytic fungi. The contrasting bacterial and fungal responses should be interpreted in the context of their ecological differences and the experimental conditions. Most soil fungi are predominantly aerobic and commonly participate in the decomposition of complex organic substrates, whereas bacterial communities include aerobic, facultatively anaerobic, and anaerobic taxa and may respond differently to readily available ${\text{NH}}_{4^{+}}$-N and ${\text{NO}}_{3^{-}}$-N. RDA and Mantel tests suggested associations of microbial community variation with EC and inorganic nitrogen, although the strength and significance of these relationships differed between bacterial and fungal communities. The contrasting textures of sandy loam and loamy sand may also have affected pore structure, water retention, aeration, and salt distribution, thereby modifying the responses of both microbial groups to BFA. The experiment was conducted using homogenized surface soil collected from the 0–20 cm layer under controlled greenhouse conditions with regular irrigation. However, actual soil moisture, oxygen status, and redox potential were not measured. Therefore, the observed differences should be regarded as combined responses to nutrient form, salinity, soil texture, and incubation conditions rather than as evidence that one microbial group was inherently more sensitive than the other.

#### 3.2.3 LEfSe analysis of soil microbial communities

**Figure 10:**
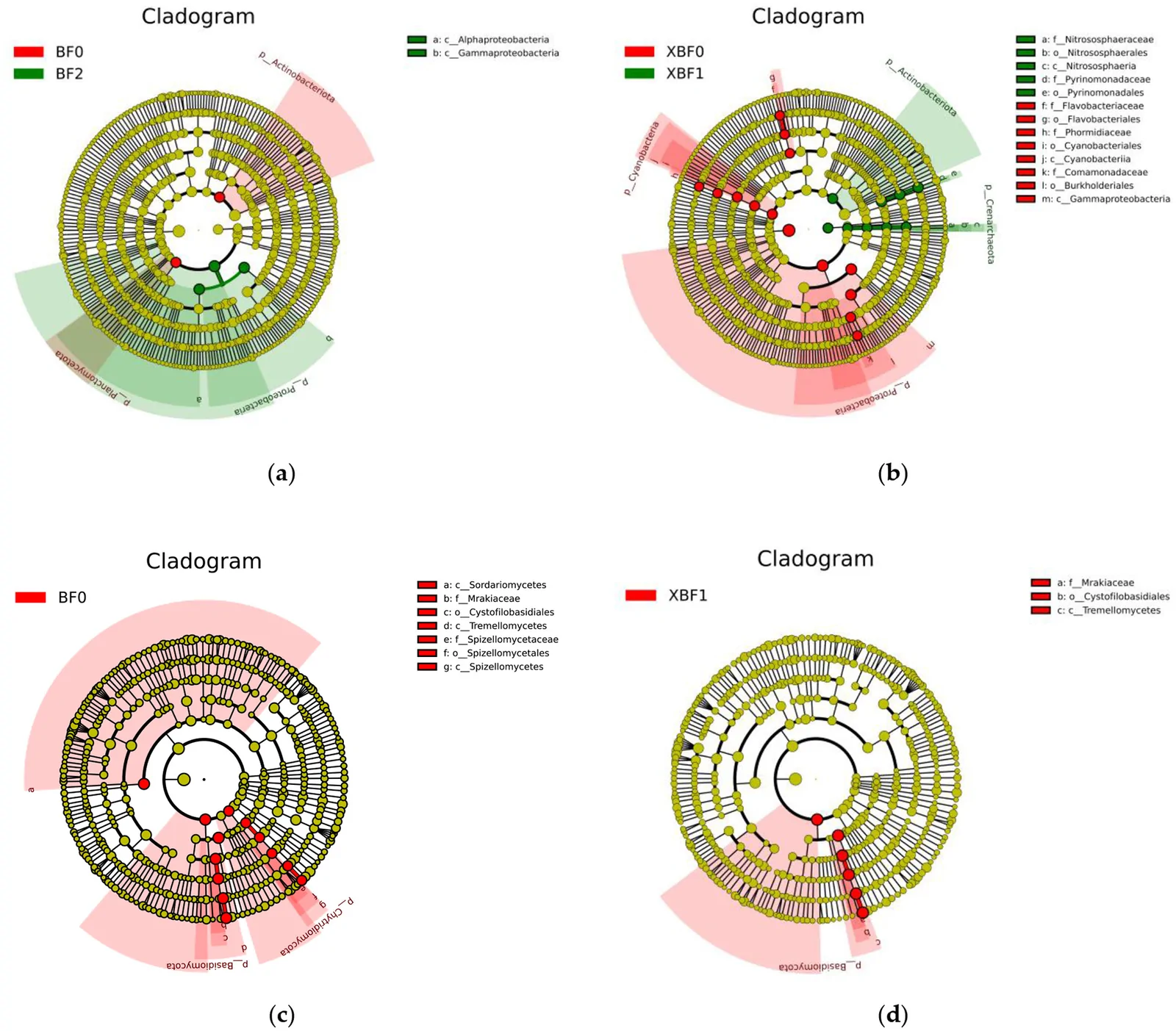
Linear discriminant analysis effect size (LEfSe) cladograms of bacterial and fungal communities in the selected treatments on day 45. **(a)** Bacterial communities in sandy loam; **(b)** bacterial communities in loamy sand; **(c)** fungal communities in sandy loam; **(d)** fungal communities in loamy sand. Nodes from the inner to outer rings represent taxa from higher to lower taxonomic ranks. Colored nodes and sectors indicate taxa significantly enriched in the corresponding treatment, whereas yellow nodes indicate taxa without significant differences. Differential taxa were identified using a Kruskal–Wallis test at *P* < 0.05 and an LDA score > 4.0 (*n* = 3 per treatment).

LEfSe analysis (Linear discriminant analysis Effect Size) can be used to compare differences among multiple sample groups and identify signature microbial communities that exhibit consistent differential features across each subgroup by integrating information from different subgroups. Clusters showing no significant differences in the present study are highlighted in yellow, whereas clusters exhibiting significant differences are labeled with colors corresponding to their respective treatment groups. Red and green represent the key microorganisms that made significant contributions within their respective treatment groups. In the sandy loam soil (Figures 10a, c), the bacterial taxa differentially enriched in the BF0 treatment included Planctomycetota and Actinobacteriota, whereas the dominant fungal group was Chytridiomycota; processing of BF2 data revealed that the dominant bacterial community was Proteobacteria and its important subphylum Gammaproteobacteria. In the loamy sand soil (Figures 10b, d), the dominant bacterial groups in the XBF0 treatment were primarily Cyanobacteria and Proteobacteria, whereas in the XBF1 treatment, the bacterial community was dominated by Actinobacteriota and Crenarchaeota, and the dominant fungal group was Basidiomycota.

Following BFA application, the dominant phyla in the sandy loam soil shifted from Actinobacteriota and Ascomycota to Proteobacteria, which is consistent with the results of phylum-level relative abundance. In the BF2 treatment, the relative abundance of the phylum Proteobacteria increased significantly, indicating enhanced dominance within the microbial community. Some members of Proteobacteria are involved in nitrogen transformation; therefore, their enrichment may have been associated with the higher inorganic nitrogen concentrations observed in the BF2 treatment. However, the present taxonomic data do not directly demonstrate enhanced biological nitrogen fixation. In loamy sand soils, the dominant prokaryotic community shifted from Proteobacteria and Cyanobacteria to Crenarchaeota and Actinobacteriota, and the fungal community was dominated by Basidiomycota. Actinomycetes are major components of soil microbial communities and are highly abundant across various ecosystems. Previous studies have demonstrated that changes in communities are closely associated with soil organic matter content and physicochemical properties, suggesting that Actinobacteriota play a significant ecological role in maintaining soil fertility and structural stability by participating in organic matter transformation processes and microbial interactions (Nemergut et al., 2010). The phylum Crenarchaeota is also a crucial group of Archaea in soil. Although minuscule, they play indispensable roles in maintaining soil fertility and ecosystem functions. They can oxidize ammonia in the soil to nitrite within the nitrogen cycle, laying the groundwork for nitrification and providing a usable nitrogen source in the soil (Tang et al., 2023). Basidiomycota are key decomposers in soil ecosystems that drive lignocellulose degradation by secreting highly efficient extracellular enzymes. They play pivotal roles in material cycling, nutrient transport, and soil aggregate formation (Lundell et al., 2010). In summary, BFA application induced distinct shifts in the microbial community structure in both soil textures, which were accompanied by changes in nutrient conditions. These taxonomic shifts coincided with changes in soil nutrient conditions; however, their functional roles in nutrient cycling and soil physical properties require direct experimental verification.

#### 3.2.4 RDA of soil microorganisms

**Figure 11:**
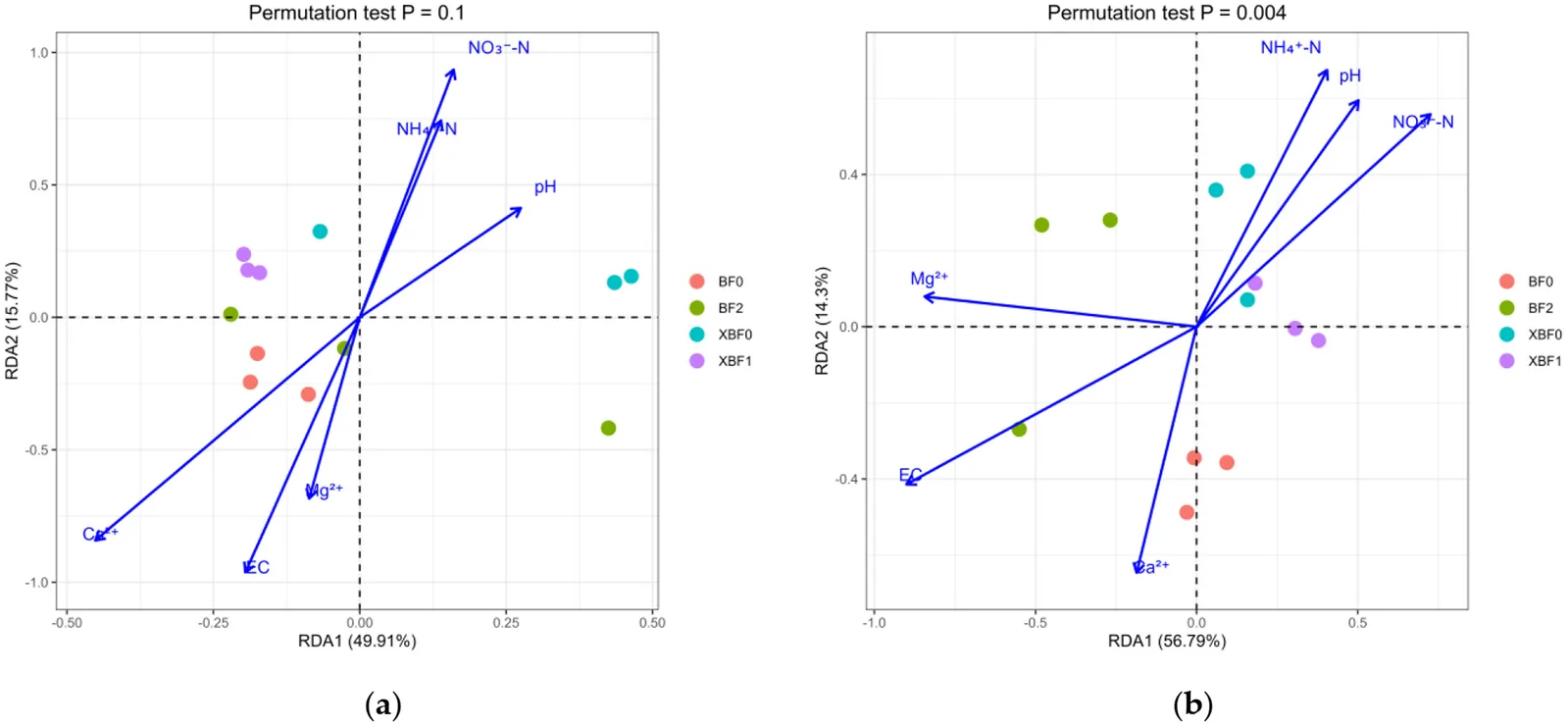
Redundancy analysis (RDA) of bacterial and fungal communities in relation to major soil environmental variables. **(a)** Bacterial communities; **(b)** fungal communities. Each point represents one biological replicate (*n* = 3), and colors indicate treatment groups. Arrows represent pH, electrical conductivity (EC), ammonium nitrogen (${\text{NH}}_{4^{+}}$-N), nitrate nitrogen (${\text{NO}}_{3^{-}}$-N), Ca^2+^, and Mg^2+^; arrow direction and length indicate the direction and relative strength of their associations with microbial community composition. Overall model significance was evaluated using 999 permutations and was not significant for bacterial communities (*P* = 0.10) but significant for fungal communities (*P* = 0.004).

The RDA (Redundancy analysis) results for bacteria and fungi in the soil are shown in Figures 11a, b, respectively. RDA1 and RDA2 explained 49.91% and 15.77% of the variation in bacterial community composition, respectively. However, the overall bacterial RDA model was not statistically significant (permutation test, *P* = 0.10), and the observed associations should therefore be interpreted as general trends. For fungal communities, RDA1 and RDA2 explained 56.79% and 14.30% of the variation, respectively, and the overall model was statistically significant (permutation test, *P* = 0.004).

The sample points for sandy loam (BF) and loamy sand (XBF) were distributed in different regions of the RDA ordination, suggesting texture-related differences in microbial community composition. Overall, among the environmental variables retained in Figure 11, EC, pH, ${\text{NH}}_{4^{+}}$-N, ${\text{NO}}_{3^{-}}$-N, Ca^2+^, and Mg^2+^ exhibited relatively long vectors, indicating comparatively strong associations with microbial community variation. EC reflects changes in the soil soluble-salt environment and osmotic conditions, whereas Ca^2+^ and Mg^2+^ are closely related to ionic balance and soil structural stability. Soil pH may shape microbial community composition by influencing nutrient availability, enzyme activity, and the physiological tolerance of different microbial taxa. ${\text{NH}}_{4^{+}}$-N and ${\text{NO}}_{3^{-}}$-N, the principal forms of inorganic nitrogen available to microorganisms, may influence microbial growth and nitrogen transformation processes. As shown in the preceding section, BFA application altered the concentrations of both forms of inorganic nitrogen, which may have contributed to the observed shifts in microbial community composition. The increase in the relative abundance of Proteobacteria in sandy loam may have been associated with the altered nutrient conditions under the BF2 treatment. However, this association does not directly demonstrate enhanced nitrogen fixation, because the functional activity of the community was not measured. In summary, bacterial and fungal community variation differed between the two soil textures and showed associations with salinity, pH, inorganic nitrogen availability, and Ca^2+^ and Mg^2+^ status. BFA-related changes in these soil properties may have contributed to the observed community shifts, with stronger statistical support for the fungal community than for the bacterial community.

#### 3.2.5 Correlation between soil environmental factors and microbial communities

**Figure 12:**
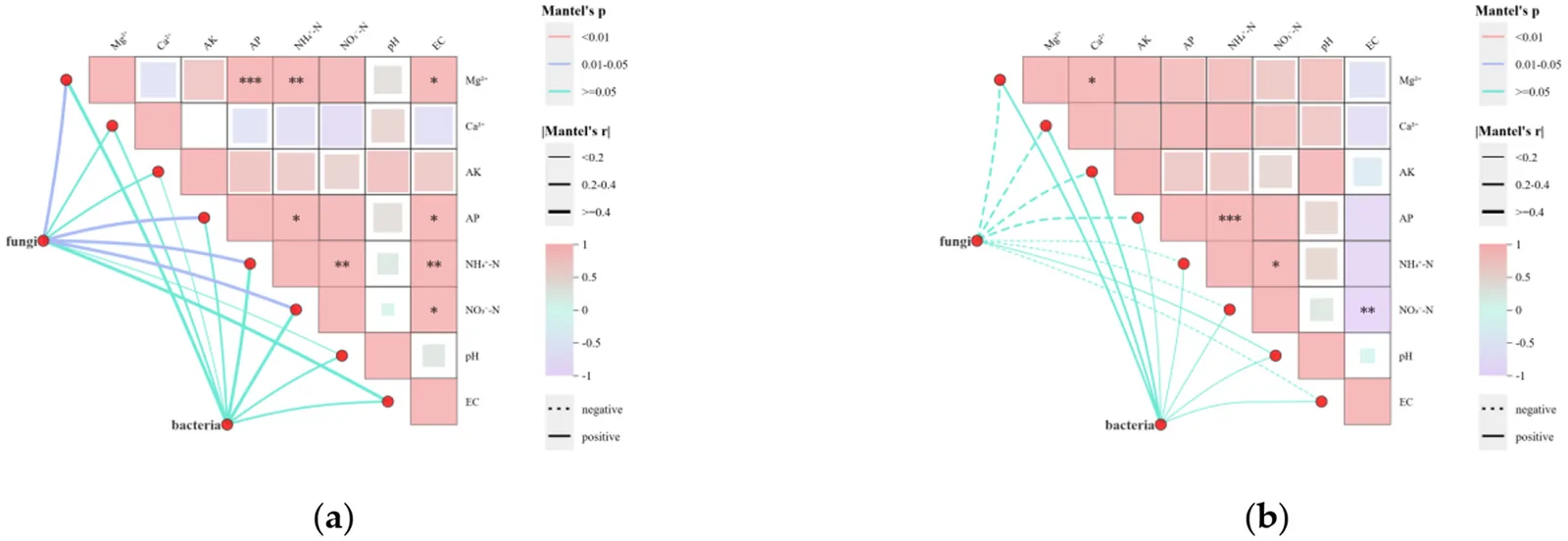
Mantel correlations between soil microbial communities and environmental variables. **(a)** Sandy loam; **(b)** loamy sand. Cell colors represent Pearson correlations among environmental variables. Lines connecting bacterial or fungal communities with environmental variables represent Mantel-test relationships; line color indicates the significance level, and line width indicates the magnitude of the Mantel correlation coefficient. Asterisks in the correlation matrix indicate statistical significance (*P < 0.05, **P < 0.01, and ***P < 0.001). Mantel-test significance was evaluated using 999 permutations. EC, electrical conductivity; ${\text{NH}}_{4^{+}}$-N, ammonium nitrogen; ${\text{NO}}_{3^{-}}$-N, nitrate nitrogen; AP, available phosphorus; AK, available potassium.

The RDA ordinations suggested associations of microbial community composition with EC, pH, ${\text{NO}}_{3^{-}}$-N, and Ca^2+^. Because the overall bacterial RDA model was not statistically significant (*P* = 0.10), the bacterial ordination patterns were interpreted only as exploratory trends. Mantel tests were subsequently used to further assess the associations between environmental variables and microbial community dissimilarity. In sandy loam soil (Figure 12a), several Mantel correlations were numerically stronger for fungal communities than for bacterial communities. However, because bacterial and fungal community dissimilarities were derived from separate 16S rRNA gene and ITS datasets, this pattern was interpreted as a difference in group-specific associations rather than direct evidence that fungi were inherently more responsive than bacteria. Among the measured variables, AP, ${\text{NH}}_{4^{+}}$-N, ${\text{NO}}_{3^{-}}$-N, and EC showed relatively closer associations with microbial communities, although the strength and significance of these relationships varied among factors. This result is generally consistent with the characteristic segregation of bacterial communities along EC and inorganic nitrogen gradients in the RDA, indicating that salinity and nitrogen may contribute to changes in the bacterial community structure in sandy loam soils. The Mantel analysis indicated an association between EC and bacterial community dissimilarity in sandy loam, while the bacterial RDA ordination showed a similar directional pattern. Electrical conductivity serves as a comprehensive indicator of soil salinity. Variation in EC not only reflects the accumulation level of soluble salt ions but also directly influences soil water availability and ion activity, thereby exerting significant regulatory effects on the microbial growth environment. Additionally, both ${\text{NH}}_{4^{+}}$-N and ${\text{NO}}_{3^{-}}$-N were associated with microbial community changes in the Mantel test, which is broadly consistent with the significant increase in nitrate and ammonium nitrogen levels observed in the BF2 treatment described earlier. The application of BFA may accelerate nitrogen cycling by promoting soil nitrogen mineralization and nitrification, possibly by providing readily available carbon sources and improving soil physicochemical conditions. Combined with the finding that the dominant bacterial phylum shifted toward Proteobacteria with nitrogen-fixing and nitrogen-metabolizing capabilities, an enhanced inorganic nitrogen supply can be inferred as one possible factor promoting the proliferation and structural reorganization of bacterial communities in sandy loam soils.

In loamy sand soils (Figure 12b), associations of bacterial community dissimilarity with ${\text{NO}}_{3^{-}}$-N and EC were observable but generally weak in the Mantel test. The directions of the EC and ${\text{NO}}_{3^{-}}$-N vectors in the bacterial RDA were broadly consistent with these patterns; however, because the overall bacterial RDA model was not significant (*P* = 0.10), these relationships were interpreted as exploratory trends. The temporal changes in inorganic nitrogen under XBF1 suggest that nitrogen availability may have contributed to microbial community differentiation during incubation. Compared with sandy loam, the association between ${\text{NH}}_{4^{+}}$-N and bacterial community dissimilarity was weaker in loamy sand, indicating a more limited or indirect relationship with this nitrogen form. In parallel, LEfSe identified Crenarchaeota as a taxon differentially enriched in XBF1. As this group includes ammonia-oxidizing archaea involved in nitrification, its enrichment may reflect changes in the soil nitrogen-transformation environment under BFA application.

The Mantel analyses showed that the strength and pattern of associations between microbial communities and environmental variables differed between sandy loam and loamy sand. Sandy loam displayed relatively stronger associations for several environmental variables, whereas most associations in loamy sand were weaker. These results indicate soil-specific patterns in the relationships between microbial communities and soil properties under BFA application.

#### 3.2.6 Microbial co-occurrence network analysis

**Figure 13:**
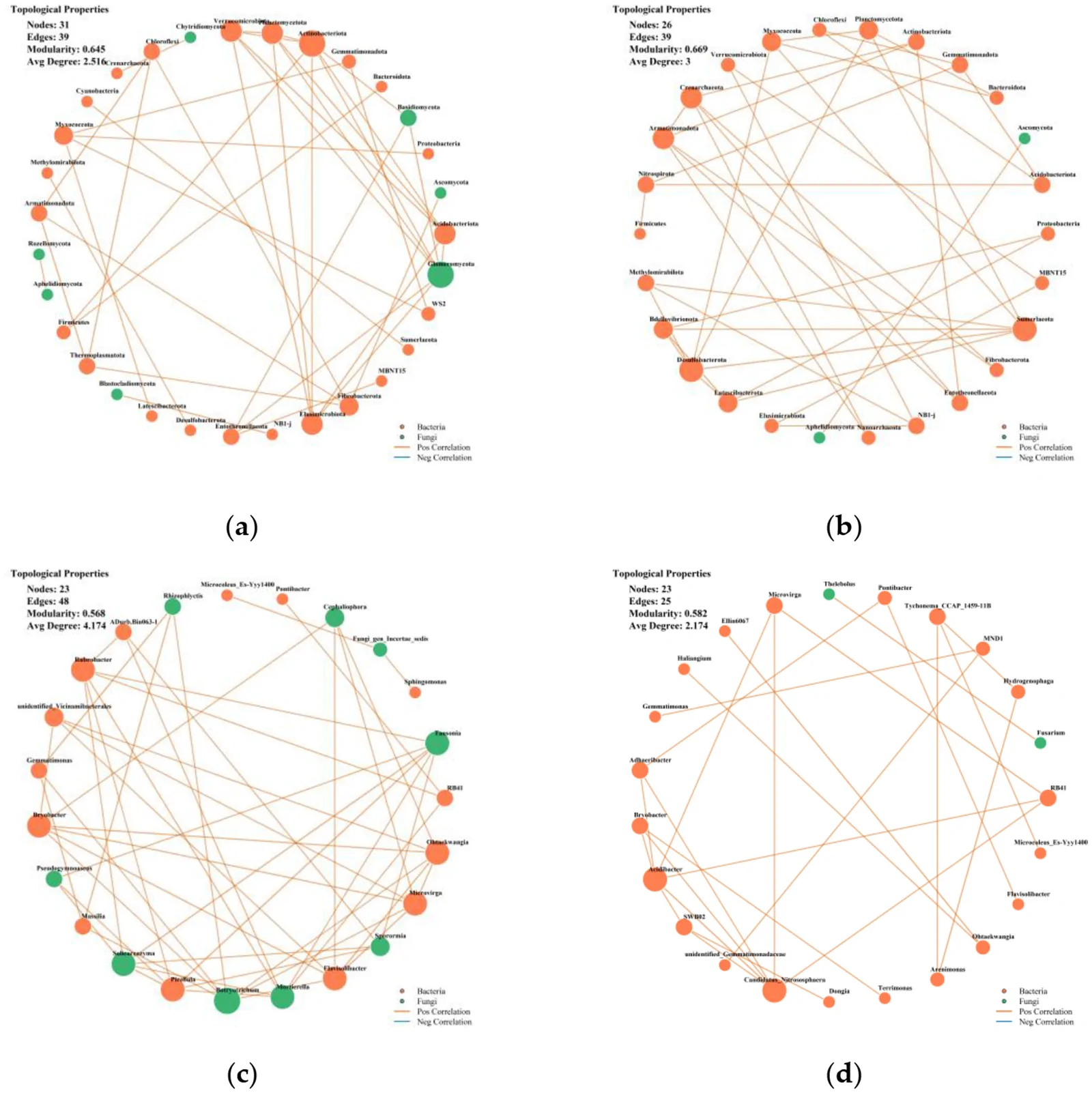
Microbial co-occurrence networks at the phylum and genus levels in sandy loam and loamy sand. **(a)** Phylum-level network in sandy loam; **(b)** phylum-level network in loamy sand; **(c)** genus-level network in sandy loam; **(d)** genus-level network in loamy sand. Nodes represent microbial taxa, with orange and green nodes representing bacterial and fungal taxa, respectively; node size is proportional to node degree. Edges represent significant Spearman correlations (|r| > 0.7, *P* < 0.05), with orange and blue edges indicating positive and negative correlations, respectively. Networks for each soil type were constructed using the selected day 45 treatments, comprising two treatments with three biological replicates each (*n* = 6 per soil type).

By constructing microbial co-occurrence networks at the phylum and genus levels, we characterized correlation patterns among microbial taxa. Network edges represented significant correlations among microbial taxa (|r| > 0.7, *P* < 0.05) rather than direct metabolic or physical interactions. In sandy loam (Figures 13a, c), the phylum-level network contained 31 nodes and 39 edges and had a modularity of 0.645, indicating a clearly modular correlation structure.

Within the phylum-level network, Glomeromycota was positively correlated with Acidobacteriota, Actinobacteriota, and Gemmatimonadota. Glomeromycota have been reported to release labile carbon compounds through their hyphal networks (Cline et al., 2018), whereas Actinobacteriota and Acidobacteriota include taxa associated with organic matter and nutrient transformation (Ward et al., 2009; Nemergut et al., 2010). The positive correlations among these taxa may reflect shared responses to soil nitrogen and phosphorus conditions rather than direct resource transfer or cooperative nutrient transformation. These correlations coincided with the higher ammonium nitrogen and available phosphorus concentrations observed in the BF2 treatment, suggesting a possible association between microbial community variation and soil nutrient availability. The genus-level network comprised 23 nodes and 48 edges, with a modularity of 0.568, and showed several cross-kingdom correlations. *Bryobacter* was positively correlated with *Pseudogymnoascus* and *Tausonia*, whereas *Pirellula* was correlated with *Solicoccozyma*. These correlations may reflect shared responses to organic-carbon availability or similar habitat preferences. However, the correlation-based network does not provide direct evidence of cooperation, functional complementarity, or sequential carbon transformation.

In the loamy sand soil (Figures 13b, d), the numbers of nodes and edges in the phylum-level network were 26 and 39, respectively, with a network modularity of 0.669 (>0.4). The genus-level network comprised 23 nodes and 25 edges, with a network modularity of 0.582 (>0.4). These results indicate that the microbial networks in loamy sand had modular correlation structures at both the phylum and genus levels. At the phylum level, Planctomycetota and Actinobacteriota were connected by a relatively strong positive edge, indicating coordinated variation in their relative abundances. Actinobacteriota include taxa associated with organic matter transformation and soil nutrient conditions (Nemergut et al., 2010). The positive correlation between these taxa suggests a possible ecological association, but does not demonstrate direct cooperation in organic matter decomposition or nutrient cycling. This correlation pattern coincided with the higher nitrate nitrogen and available phosphorus concentrations observed in the XBF1 treatment; however, it does not establish a causal relationship between microbial associations and nutrient availability. At the phylum level, Chloroflexi, Verrucomicrobiota, and Sumerlaeota formed a closely connected cluster in the loamy sand network. These taxa have previously been associated with carbon transformation and adaptation to nutrient-limited environments (Naziebło et al., 2026; Sichert et al., 2020; Skoog and Bosak, 2024). Nevertheless, their positive correlations in the present study indicate coordinated abundance patterns rather than confirmed synergistic carbon utilization. At the genus level, *Candidatus Nitrososphaera* was a highly connected node associated with *Acidibacter, MBNT15*, and *Ellin6067*. *Candidatus Nitrososphaera* is an ammonia-oxidizing archaeal genus involved in the conversion of ${\text{NH}}_{4}^{+}$ to ${\text{NO}}_{2}^{-}$ during nitrification (Tourna et al., 2011). Its correlations with other taxa may therefore be associated with variation in soil nitrogen conditions, although direct metabolic coupling was not examined. Acidibacter is affiliated with Acidobacteriota, and its abundance has been associated with soil organic matter and nitrogen conditions (Jiang et al., 2024). These correlations may reflect coordinated responses to carbon and nitrogen availability. However, the present network analysis does not demonstrate direct metabolic coupling among these taxa or establish a “carbon production–utilization–decomposition” mechanism.

Overall, positive correlations were more frequent than negative correlations in the microbial networks of both soil types. These patterns suggest coordinated abundance changes or shared responses to environmental conditions but do not provide direct evidence of ecological or metabolic interactions.

## 4 Conclusions

Application of biochemical fulvic acid (BFA) produced soil-specific changes in soil physicochemical properties and nutrient availability that varied among application rates. Relative to the control, BFA temporarily alleviated the decline in soil pH during the early incubation stage, whereas soil EC decreased under low application rates but increased under higher rates. Meanwhile, inorganic nitrogen, available phosphorus, available potassium, and Mg^2+^ generally showed dose-dependent responses, with moderate BFA application rates producing the most favorable overall effects. Based on the evaluated indicators, BF2 in sandy loam and XBF1 in loamy sand produced the most favorable overall responses under the conditions of this incubation experiment.

At the selected soil-specific BFA rates, microbial diversity and community composition showed different responses in the two soils. Specifically, the relative abundances of Proteobacteria and Actinobacteriota increased in sandy loam, whereas those of Acidobacteriota and Actinobacteriota increased and that of Proteobacteria decreased in loamy sand; the relative abundance of Ascomycota increased in both soils.

Redundancy analysis and Mantel tests associated microbial community variation mainly with pH, electrical conductivity, Ca^2+^, Mg^2+^, and inorganic nitrogen. Furthermore, co-occurrence networks were dominated by positive correlations, suggesting coordinated abundance patterns under BFA application rather than direct metabolic interactions.

These findings highlight the potential of BFA to improve nutrient availability and modify microbial community composition in sodic saline–alkali soils. However, because the microbial analysis was conducted on selected treatments on day 45, future studies should include longer-term validation and comparisons with a range of organic and inorganic amendments to fully evaluate its agronomic and economic benefits.

## Data Availability

The raw bacterial 16S rRNA gene and fungal ITS amplicon sequencing reads generated in this study have been deposited in the NCBI Sequence Read Archive (SRA) under BioProject accession number PRJNA1490088 and SRA Study accession number SRP715342. The 24 sequencing runs are available under accession numbers SRR39465635-SRR39465658. The data are publicly available through the NCBI BioProject record at https://www.ncbi.nlm.nih.gov/bioproject/PRJNA1490088.
